# HMGB2 Promotes Cardiomyocyte Proliferation and Heart Regeneration Through MTA2‐Driven Metabolic Reprogramming

**DOI:** 10.1002/advs.202505820

**Published:** 2025-10-15

**Authors:** Liu‐Hua Zhou, Ling‐Feng Gu, Yi‐Xi Chen, Peng Jing, Tong‐Tong Yang, Ye He, Yu‐Lin Bao, Xiang‐Zheng Zhang, Chong Du, Si‐Bo Wang, Tian‐Kai Shan, Jia‐Yi Chen, Hao Wang, Qi‐Ming Wang, Li‐Ping Xie, Yang Zhao, Yong Ji, Lian‐Sheng Wang

**Affiliations:** ^1^ Department of Cardiology the First Affiliated Hospital with Nanjing Medical University Nanjing 210029 China; ^2^ State Key Laboratory of Reproductive Medicine and Offspring Health Department of Histology and Embryology Nanjing Medical University Nanjing 211166 China; ^3^ Key Laboratory of Cardiovascular and Cerebrovascular Medicine Key Laboratory of Targeted Intervention of Cardiovascular Disease Collaborative Innovation Center for Cardiovascular Disease Translational Medicine Nanjing Medical University Nanjing 211166 China; ^4^ Department of Biostatistics School of Public Health China International Cooperation Center for Environment and Human Health Nanjing Medical University Nanjing 211166 China

**Keywords:** cardiomyocyte proliferation, glucose metabolism, heart regeneration, HMGB2, MTA2

## Abstract

The neonatal heart possesses the unique ability to regenerate post‐injury. Underlying related mechanisms and reactivation of this process are crucial for regeneration medicine. Using quantitative proteomics with tandem mass tag labeling, RNA‐sequencing (RNA‐seq) and single‐nucleus RNA‐seq dataset analyses, high mobility group box 2 (HMGB2) is identified as a key regulator of cardiomyocyte proliferation, whose expression declines during postnatal heart development and increases in the high regenerative potential cardiomyocyte populations in hearts post‐injury. Cardiomyocyte‐specific HMGB2 knockdown curtails cardiomyocyte proliferation and impairs heart regeneration following apical resection in neonatal mice, while cardiomyocyte‐specific HMGB2 overexpression enhances cardiomyocyte proliferation and facilitates cardiac regeneration and repair in adult mice post‐myocardial infarction. Mechanistically, RNA‐seq analysis reveals that HMGB2 promotes cardiomyocyte proliferation via activating hypoxia inducible factor 1ɑ (HIF‐1α)‐mediated glycolysis. This study further finds HMGB2 can directly interact with metastasis‐associated protein 2 (MTA2) and inhibit its ubiquitination degradation to stabilize HIF‐1α protein through immunoprecipitation‐mass spectrometry (IP‐MS) analysis. Finally, overexpression of HIF‐1α or MTA2 also promotes cardiomyocyte proliferation and cardiac repair in adult mice following MI. Taken together, these findings highlight that HMGB2 plays a crucial role in promoting heart regeneration through regulating glycolysis. Activating the HMGB2‐MTA2‐HIF‐1α axis might serve as a potential therapeutic option for regenerative therapies post‐myocardial injury.

## Graphical abstract

In contrast to the adult mammalian heart, which lacks regenerative capacity, the neonatal heart retains a transient regenerative capacity. In this study, high mobility group box 2 (HMGB2) is identified as a crucial protein driving heart regeneration based on comprehensive analyses using quantitative proteomics with tandem mass tag (TMT) labelling, RNA‐sequencing (RNA‐seq), and a single‐nucleus RNA‐seq database. Through a combination of in vivo and in vitro experiments, it is demonstrated that cardiomyocyte‐specific overexpression of HMGB2 significantly enhance s cardiomyocyte proliferation and promotes cardiac repair after myocardial injury, whereas cardiomyocyte‐specific knockdown of HMGB2 produces the opposite effect. Mechanistically, HMGB2 is found to directly interact with metastasis‐associated protein 2 (MTA2) and inhibit its ubiquitination degradation to stabilize hypoxia inducible factor 1α (HIF‐1α), thereby activating glycolysis through RNA‐seq and immunoprecipitation mass spectrometry (IP‐MS) analysis. These findings highlight the pivotal role of HMGB2 in driving cardiomyocyte proliferation and heart regeneration via metabolic reprogramming, which provides novel avenues for developing targeted therapies aimed at improving cardiac regeneration and repair following myocardial injury.

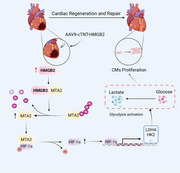

## 1 Introduction

Ischemic heart disease is a leading cause of cardiovascular morbidity and mortality worldwide.^[^ ^1^, ^2^ ^]^ Current treatments for myocardial infarction (MI) primarily focus on restoring blood flow to the infarcted coronary artery and improving myocardial perfusion, rather than fundamentally resolving the replenishment of dying cardiomyocytes or the repair of necrotic myocardium.^[^ ^3^, ^4^, ^5^, ^6^ ^]^ Therefore, addressing these aspects might further improve the long‐term outcomes of patients with acute myocardial infarction. It is established that newborn mice can achieve complete functional and morphological heart repair through endogenous regeneration following apical resection (AR). However, this capacity rapidly diminishes within seven days after birth.^[^ ^7^, ^8^, ^9^ ^]^ Elucidating the mechanisms of neonatal heart regeneration and identifying effective molecular targets to reactivate the cell cycle of mature cardiomyocytes may provide valuable strategies and insights for treating myocardial injury.

As a member of the high mobility group box (HMGB) protein family, high mobility group box 2 (HMGB2) is one of the earliest identified and most ubiquitously expressed proteins across eukaryotes.^[^ ^10^, ^11^ ^]^ Accumulating studies have recognized HMGB2 as a pivotal regulatory factor in gene expression and key cellular functions, including cell proliferation, apoptosis, and autophagy.^[^ ^11^, ^12^ ^]^ Previous research has revealed that HMGB2 can promote the upregulation of *Myf5* and *Cyclin A2*, thereby regulating satellite‐cell‐mediated skeletal muscle regeneration.^[^ ^13^ ^]^ Additionally, HMGB2 was found to be a key regulator of cell proliferation and hepatocyte size during liver regeneration.^[^ ^14^ ^]^ Studies have also found that HMGB2 plays a crucial role in regulating platelet activation and cardiac function in cardiovascular diseases.^[^ ^15^, ^16^, ^17^, ^18^ ^]^ For example, HMGB2 deficiency may impair cardiac function by downregulating SERCA2a expression after transverse aortic constriction.^[^ ^18^ ^]^ However, the role of HMGB2 in cardiomyocyte proliferation and heart regeneration post‐injury is not fully understood.

In this study, we demonstrated that the expression pattern of HMGB2 correlated with the decline in regenerative capacity of mouse hearts. Under physiological conditions, overexpression of HMGB2 enhanced cardiomyocyte proliferation. We further found that knockdown of HMGB2 impaired heart regeneration after AR in neonatal mice, while HMGB2 overexpression promoted cardiomyocyte proliferation and cardiac repair after MI in adult mice (56‐day‐old mice). RNA‐seq analysis revealed that HMGB2 promoted cardiomyocyte proliferation by activating glycolysis. We further discovered that MTA2 served as a direct downstream target of HMGB2, activating the HIF‐1α‐mediated glycolysis through IP/MS analysis. Therefore, these functional and mechanistic studies identified HMGB2 as a critical regulator of cardiomyocyte proliferation, highlighting its therapeutic potential in heart regeneration.

## 2 Results

### 2.1 HMGB2 is Involved in Heart Regeneration in Mice

**Figure 1:**
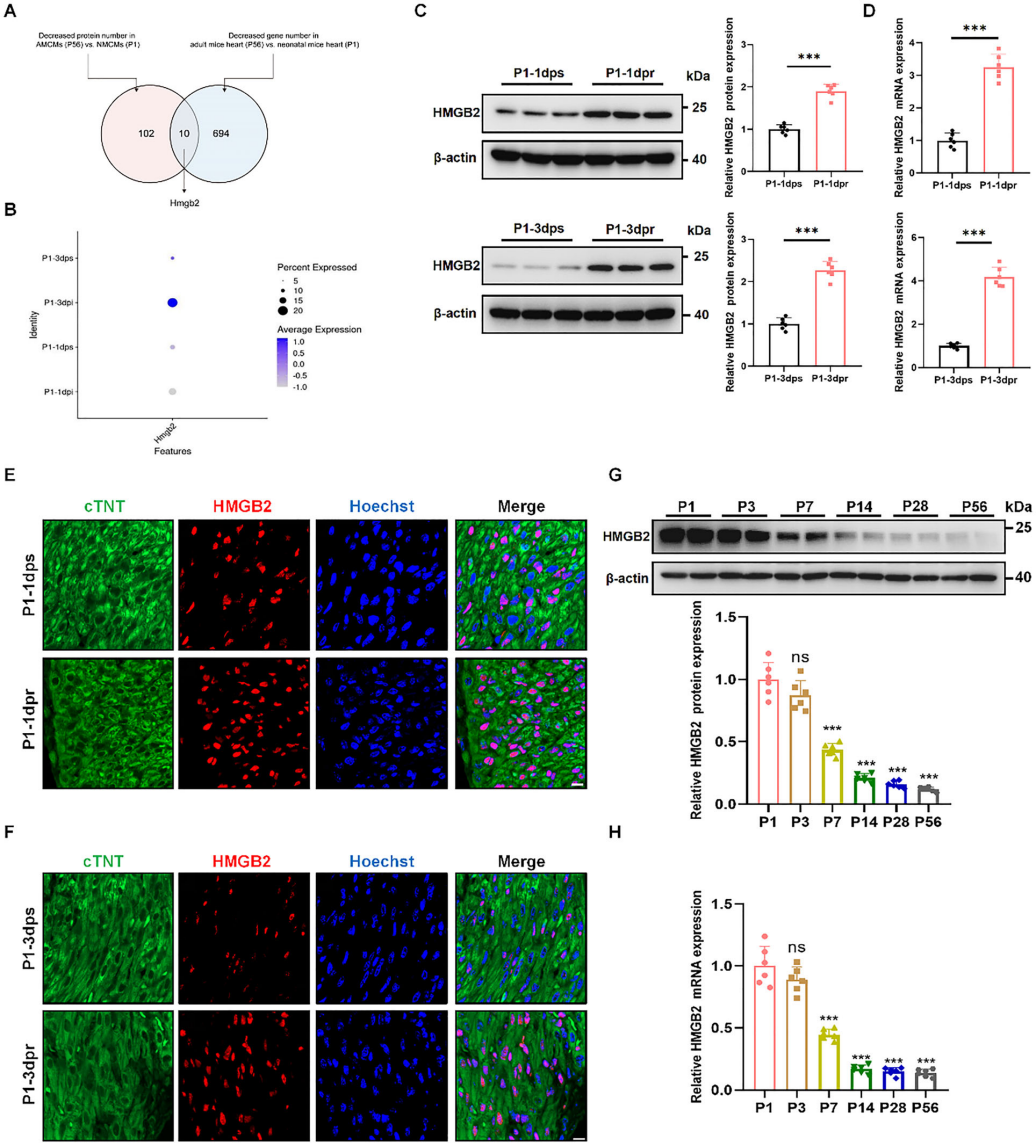
Identification of HMGB2 as a vital protein involved in neonatal heart regeneration. A, Venn diagram showing 10 candidates by cross‐comparing these differentially downregulated genes with proteins. Red represents the differentially downregulated proteins between adult mouse cardiomyocytes (AMCMs) and neonatal mouse cardiomyocytes (NMCMs). Blue represents the differentially downregulated genes between adult mouse hearts and neonatal mouse hearts. Differentially downregulated proteins and genes were identified when adjusted P value < 0.05 and Log_2_FC < ‐2.5. B, Single‐nucleus RNA‐sequencing analysis of HMGB2 expression in regenerative hearts 1 and 3 days after MI or sham surgery at P1 mice from the GEO repository (GSE130699). dps: day post‐sham. dpi: day post‐infarction. C, Western blot and quantification of HMGB2 protein expression in the sham mouse myocardium and apical resection (AR) mouse myocardium (n=6 each). D, Quantitative reverse‐transcription polymerase chain reaction (qRT‐PCR) analysis of HMGB2 mRNA expression in the sham mouse myocardium and AR mouse myocardium (n=6 each). E, Immunofluorescence (IF) staining analysis of HMGB2 expression in the sham mouse myocardium and AR mouse myocardium at 1 dpr. cTNT (green), HMGB2 (red), Hoechst (blue). scale bar, 10 µm. F, IF staining analysis of HMGB2 expression in the sham mouse myocardium and AR mouse myocardium at 3 dpr. cTNT (green), HMGB2 (red), Hoechst (blue). scale bar, 10 µm. G, Western blot and quantification of HMGB2 protein expression at different time points in mouse myocardium (n=6 each). H, qRT‐PCR analysis of HMGB2 mRNA expression at different time points in mouse myocardium (n=6 each). Statistical analysis was performed by an unpaired Student's *t*‐test for C‐D. Statistical analysis was performed by one‐way ANOVA followed by Tukey multiple comparisons for G‐H. Ns, not significant. ^***^ *P*<0.001.

To investigate the molecular mechanisms regulating heart regeneration, we first isolated neonatal mouse cardiomyocytes (NMCMs) and adult mouse cardiomyocytes (AMCMs), respectively. Using quantitative proteomics with tandem mass tag labeling analysis between AMCMs and NMCMs, a total of 283 differentially expressed proteins (DEPs) were identified (Figure  S1A,B, Supporting Information). Subsequently, we used RNA‐sequencing (RNA‐seq) analysis of the hearts of postnatal day 56 (P56) and P1 mice and found 435 upregulated genes and 704 downregulated genes from the GEO repository (GSE213233) (Figure S1C,D, Supporting Information). By cross‐comparing these differentially downregulated genes with proteins, 10 candidates were significantly identified. Based on the following criteria: 1) substantial differential expression between groups (as determined by P value), 2) high evolutionary conservation across species, and 3) known involvement in regulating regeneration and cell proliferation, HMGB2 was selected for further investigation in cardiomyocyte proliferation and heart regeneration (**Figure** **1**A; Table S1, Supporting Information). Furthermore, we analyzed a single‐nucleus RNA‐seq dataset (GSE130699) and found HMGB2 was significantly upregulated through analysis of cardiomyocyte populations in regenerative hearts 1 and 3 days after MI or sham surgery in P1 mice (Figure 1B).

Next, we found that HMGB2 was primarily expressed in cardiomyocytes (Figure S1E,F, Supporting Information). To further explore the involvement of HMGB2 in heart regeneration, we detected the expression of HMGB2 at various time points after AR in P1 mice. Western blot and quantitative reverse transcription‐polymerase chain reaction (qRT‐PCR) results showed the expression of HMGB2 was significantly increased in both cardiomyocytes and myocardial tissues at 1 and 3 dpr (Figure 1C,D; Figure S1G,H, Supporting Information). Meanwhile, immunofluorescence (IF) staining demonstrated widespread HMGB2 activation in the border zone of the neonatal heart after myocardial injury (Figure 1E,F).

Additionally, we examined HMGB2 expression in the ventricular myocardium of mice at various postnatal stages. Western blot and qRT‐PCR results showed that HMGB2 expression peaked at P1, then significantly declined at P7, and remained low until P56 (Figure 1G,H). These results were consistent with the findings of IF staining analysis (Figure S1I, Supporting Information). In summary, these findings suggest that HMGB2 may play a vital function in heart regeneration.

### 2.2 HMGB2 is Crucial for Cardiomyocyte Proliferation In Vitro

**Figure 2:**
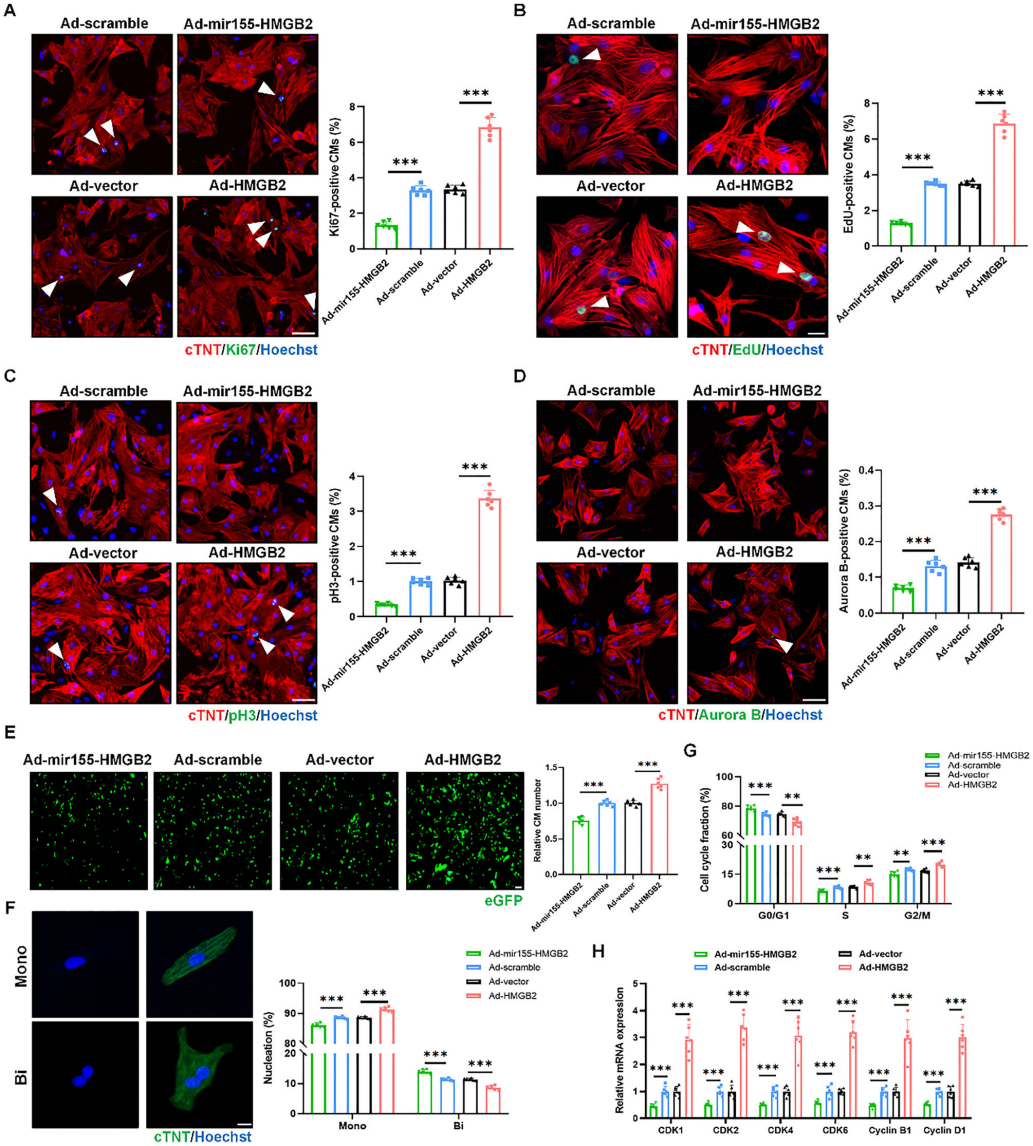
HMGB2 is essential for the proliferation of neonatal mouse cardiomyocytes in vitro. A, Ki67 immunofluorescence staining and quantification of Ki67‐positive CMs in NMCMs transfected with Ad5‐cTNT‐mir155‐HMGB2, Ad5‐cTNT‐mir155‐scramble, Ad5‐cTNT vector, or Ad5‐cTNT‐HMGB2 for 24 h (n=6 each). cTNT (red), Ki67 (green), Hoechst (blue). scale bar, 50 µm. B, EdU immunofluorescence staining and quantification of EdU‐positive CMs in NMCMs transfected with Ad5‐cTNT‐mir155‐HMGB2, Ad5‐cTNT‐mir155‐scramble, Ad5‐cTNT vector, or Ad5‐cTNT‐HMGB2 for 24 h (n=6 each). cTNT (red), EdU (green), Hoechst (blue). scale bar, 20 µm. C, pH3 immunofluorescence staining and quantification of pH3‐positive CMs in NMCMs transfected with Ad5‐cTNT‐mir155‐HMGB2, Ad5‐cTNT‐mir155‐scramble, Ad5‐cTNT vector, or Ad5‐cTNT‐HMGB2 for 24 h (n=6 each). cTNT (red), pH3 (green), Hoechst (blue). scale bar, 50 µm. D, Aurora B immunofluorescence staining and quantification of Aurora B‐positive CMs in NMCMs transfected with Ad5‐cTNT‐mir155‐HMGB2, Ad5‐cTNT‐mir155‐scramble, Ad5‐cTNT vector, or Ad5‐cTNT‐HMGB2 for 24 h (n=6 each). cTNT (red), Aurora B (green), Hoechst (blue). scale bar, 50 µm. E, eGFP representative images and quantification of CM number in NMCMs transfected with Ad5‐cTNT‐mir155‐HMGB2, Ad5‐cTNT‐mir155‐scramble, Ad5‐cTNT vector, or Ad5‐cTNT‐HMGB2 for 24 h (n=6 each). eGFP (green). scale bar, 20 µm. F, Representative images and quantification of CM nucleation in NMCMs transfected with Ad5‐cTNT‐mir155‐HMGB2, Ad5‐cTNT‐mir155‐scramble, Ad5‐cTNT vector, or Ad5‐cTNT‐HMGB2 for 24 h (n=6 each). cTNT (green), Hoechst (blue). scale bar, 20 µm. G, Flow cytometry detection of cell cycle alterations in NMCMs transfected with Ad5‐cTNT‐mir155‐HMGB2, Ad5‐cTNT‐mir155‐scramble, Ad5‐cTNT vector, or Ad5‐cTNT‐HMGB2 for 24 h (n=6 each). H, qRT‐PCR analysis of cell cycle‐related genes expression in NMCMs transfected with Ad5‐cTNT‐mir155‐HMGB2, Ad5‐cTNT‐mir155‐scramble, Ad5‐cTNT vector, or Ad5‐cTNT‐HMGB2 for 24 h (n=6 each). Statistical analysis was performed by an unpaired Student's *t*‐test for A‐H. ^**^ *P*<0.01; ^***^ *P*<0.001.

To further investigate the role of HMGB2 in cardiomyocyte proliferation, we constructed cardiomyocyte‐specific adenoviruses carrying HMGB2 interference sequence (Ad5‐cTNT‐mir155‐HMGB2), cardiomyocyte‐specific adenoviruses carrying HMGB2 coding sequence (Ad5‐cTNT‐HMGB2), and control vectors (Ad5‐cTNT‐mir155‐scramble and Ad5‐cTNT vector). Through infection with varying multiplicities of infection (MOI), we determined that the optimal infection concentration was 100 (Figure S2A, Supporting Information). Adenovirus transfection efficiency was assessed via Western blot and qRT‐PCR (Figure S2B–E, Supporting Information). Next, we used IF staining to evaluate the effect of HMGB2 on the proliferation of NMCMs. Results showed that HMGB2 overexpression promoted cardiomyocyte proliferation, as indicated by increased numbers of cell cycle activity (Ki67^+^), DNA synthesis (EdU^+^), mitosis (pH3^+^), and cytoplasmic division (Aurora B^+^) cardiomyocytes, while HMGB2 knockdown significantly inhibited cardiomyocyte proliferation (**Figure** **2**A–D; Figure S3A–D, Supporting Information). Quantitative analysis showed that HMGB2 overexpression raised cardiomyocyte number, while HMGB2 knockdown reduced it (Figure 2E). Additionally, HMGB2 overexpression increased the proportion of mononucleated cardiomyocytes and decreased the proportion of binucleated cardiomyocytes. Conversely, HMGB2 knockdown produced the opposite effect (Figure 2F). Flow cytometry analysis revealed that HMGB2 overexpression led to an accumulation of cardiomyocytes in the S and G2/M phases, while HMGB2 knockdown resulted in an accumulation of cardiomyocytes in the G0/G1 phases (Figure 2G). Further validation was conducted on the expression levels of proliferation‐related genes, which showed that mRNA levels of CDK1, CDK2, CDK4, CDK6, Cyclin D1, and Cyclin B1 were significantly increased following HMGB2 overexpression and decreased after HMGB2 knockdown (Figure 2H). Moreover, to investigate the effect of HMGB2 on cardiomyocyte apoptosis, we conducted IF staining and found that neither overexpression nor knockdown of HMGB2 significantly affected cardiomyocyte apoptosis (Figure S4A, Supporting Information). We further isolated cardiomyocytes from adult mouse hearts. IF staining for cTNT and Ki67, EdU, or pH3 revealed a significant increase in the proliferation of AMCMs following HMGB2 overexpression (Figure S5A–E, Supporting Information). These data suggest that HMGB2 substantially enhances the proliferation potential of cardiomyocytes.

### 2.3 HMGB2 is Required for Heart Regeneration in Neonatal Mice

**Figure 3:**
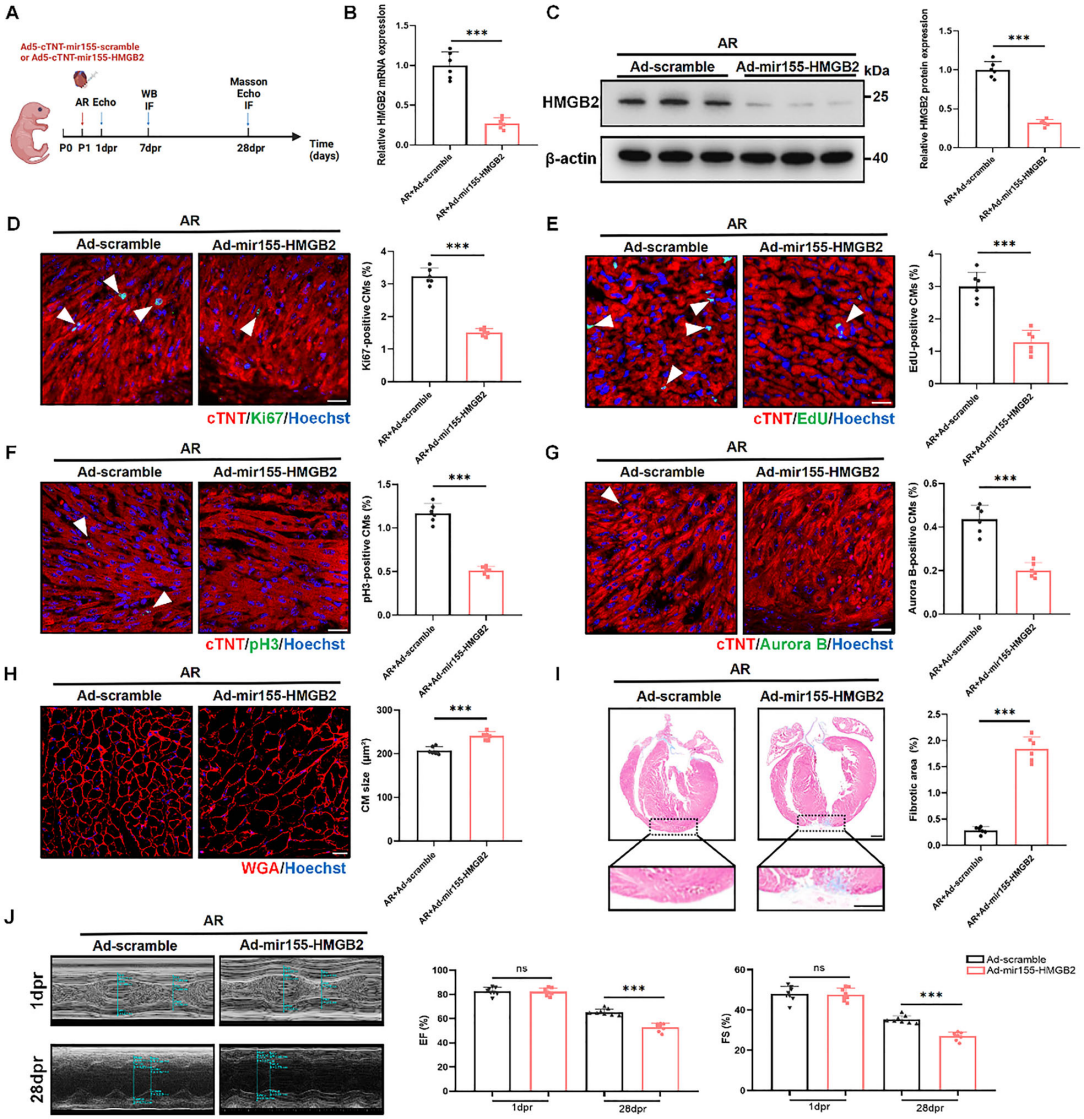
HMGB2 knockdown inhibits heart regeneration in neonatal mice after apical resection. A, Schematic representation of injecting Ad5‐cTNT‐mir155‐scramble or Ad5‐cTNT‐mir155‐HMGB2 into the resection border zone of P1 mice after apical resection (Created in https://BioRender.com). B, qRT‐PCR analysis of HMGB2 mRNA expression in mice injected with Ad5‐cTNT‐mir155‐scramble or Ad5‐cTNT‐mir155‐HMGB2 at 7 dpr (n=6 each). C, Western blot and quantification of HMGB2 protein expression in mice injected with Ad5‐cTNT‐mir155‐scramble or Ad5‐cTNT‐mir155‐HMGB2 at 7 dpr (n=6 each). D, Ki67 immunofluorescence staining and quantification of Ki67‐positive CMs in mice injected with Ad5‐cTNT‐mir155‐scramble or Ad5‐cTNT‐mir155‐HMGB2 at 7 dpr (n=6 each). cTNT (red), Ki67 (green), Hoechst (blue). scale bar, 20 µm. E, EdU immunofluorescence staining and quantification of EdU‐positive CMs in mice injected with Ad5‐cTNT‐mir155‐scramble or Ad5‐cTNT‐mir155‐HMGB2 at 7 dpr (n=6 each). cTNT (red), EdU (green), Hoechst (blue). scale bar, 20 µm. F, pH3 immunofluorescence staining and quantification of pH3‐positive CMs in mice injected with Ad5‐cTNT‐mir155‐scramble or Ad5‐cTNT‐mir155‐HMGB2 at 7 dpr (n=6 each). cTNT (red), pH3 (green), Hoechst (blue). scale bar, 20 µm. G, Aurora B immunofluorescence staining and quantification of Aurora B‐positive CMs in mice injected with Ad5‐cTNT‐mir155‐scramble or Ad5‐cTNT‐mir155‐HMGB2 at 7 dpr (n=6 each). cTNT (red), Aurora B (green), Hoechst (blue). scale bar, 20 µm. H, WGA immunofluorescence staining and quantification of CM size in mice injected with Ad5‐cTNT‐mir155‐scramble or Ad5‐cTNT‐mir155‐HMGB2 at 28 dpr (n=6 each). WGA (red), Hoechst (blue). scale bar, 20 µm. I, Representative Masson's trichrome staining and quantification of fibrotic area in mice injected with Ad5‐cTNT‐mir155‐scramble or Ad5‐cTNT‐mir155‐HMGB2 at 28 dpr (n=6 each). scale bar, 500 µm. J, Echocardiography analysis of the left ventricle ejection fraction (EF) and fractional shortening (FS) in mice injected with Ad5‐cTNT‐mir155‐scramble or Ad5‐cTNT‐mir155‐HMGB2 at 1 dpr and 28 dpr (n=8 each). Statistical analysis was performed by an unpaired Student's *t*‐test for B‐J. Ns, not significant. ^***^ *P*<0.001.

To elucidate the role of HMGB2 in heart regeneration, we constructed an AR model in P1 mice. Immediately following the procedure, we injected Ad5‐cTNT‐mir155‐HMGB2 or Ad5‐cTNT‐mir155‐scramble into the resection border zone of P1 mouse hearts (**Figure** **3**A). The expression of HMGB2 was examined in both sham groups and AR groups, confirming successful intervention (Figure 3B,C; Figure S6A–C, Supporting Information). In the sham groups, HMGB2 knockdown reduced the proportion of Ki67^+^, EdU^+^, pH3^+^, and Aurora B^+^ cardiomyocytes compared to mice treated with Ad5‐cTNT‐mir155‐scramble (Figure S6D–G, Supporting Information). In the AR groups, IF staining revealed a significant decrease in cardiomyocyte proliferation following HMGB2 knockdown (Figure 3D–G). Additionally, cardiomyocyte size was evaluated using wheat germ agglutinin (WGA) staining, which showed a significantly larger cross‐sectional area in HMGB2 knockdown mice from both sham and AR groups compared to mice treated with Ad5‐cTNT‐mir155‐scramble (Figure 3H; Figure S6H, Supporting Information). Echocardiographic and histological analysis performed 28 days after AR revealed that HMGB2 knockdown significantly impaired cardiac function and inhibited heart regeneration in the AR groups (Figure 3I,J). In contrast, HMGB2 knockdown had no significant effect on cardiac function or gross cardiac morphology in the sham groups (Figure S6I,J, Supporting Information). Taken together, these findings suggest that HMGB2 is essential for cardiomyocyte proliferation during neonatal heart regeneration post‐injury.

### 2.4 HMGB2 Overexpression Prolongs the Cardiomyocyte Proliferative Window

To determine whether HMGB2 could extend the window of heart regeneration, we constructed a cardiomyocyte‐specific adeno‐associated virus 9 carrying HMGB2 (AAV9‐cTNT‐HMGB2) or a control vector (AAV9‐cTNT vector) and injected them into the myocardium of P1 mice (Figure S7A, Supporting Information). Successful viral intervention was confirmed through Western blot and qRT‐PCR at various stages (Figure S7B–F, Supporting Information). IF analysis revealed that HMGB2 overexpression increased the proportion of Ki67^+^, EdU^+^, pH3^+^, and Aurora B^+^ cardiomyocytes and the total number of cardiomyocytes, and decreased cardiomyocyte size (Figure S8A–F, Supporting Information). Additionally, HMGB2 overexpression resulted in a higher number of mononucleated cardiomyocytes with decreased ploidy compared to the control vector group (Figure S8G, Supporting Information). Histological and echocardiographic analysis at P28 showed that HMGB2 overexpression did not significantly affect cardiac function or induce pathological cardiac hypertrophy (Figure S8H–L, Supporting Information). These results suggest that HMGB2 overexpression enhances the proliferative capacity of cardiomyocytes without causing pathological hypertrophy or impairing cardiac function under physiological conditions.

### 2.5 HMGB2 Promotes Cardiac Regeneration and Repair in Adult Mice

**Figure 4:**
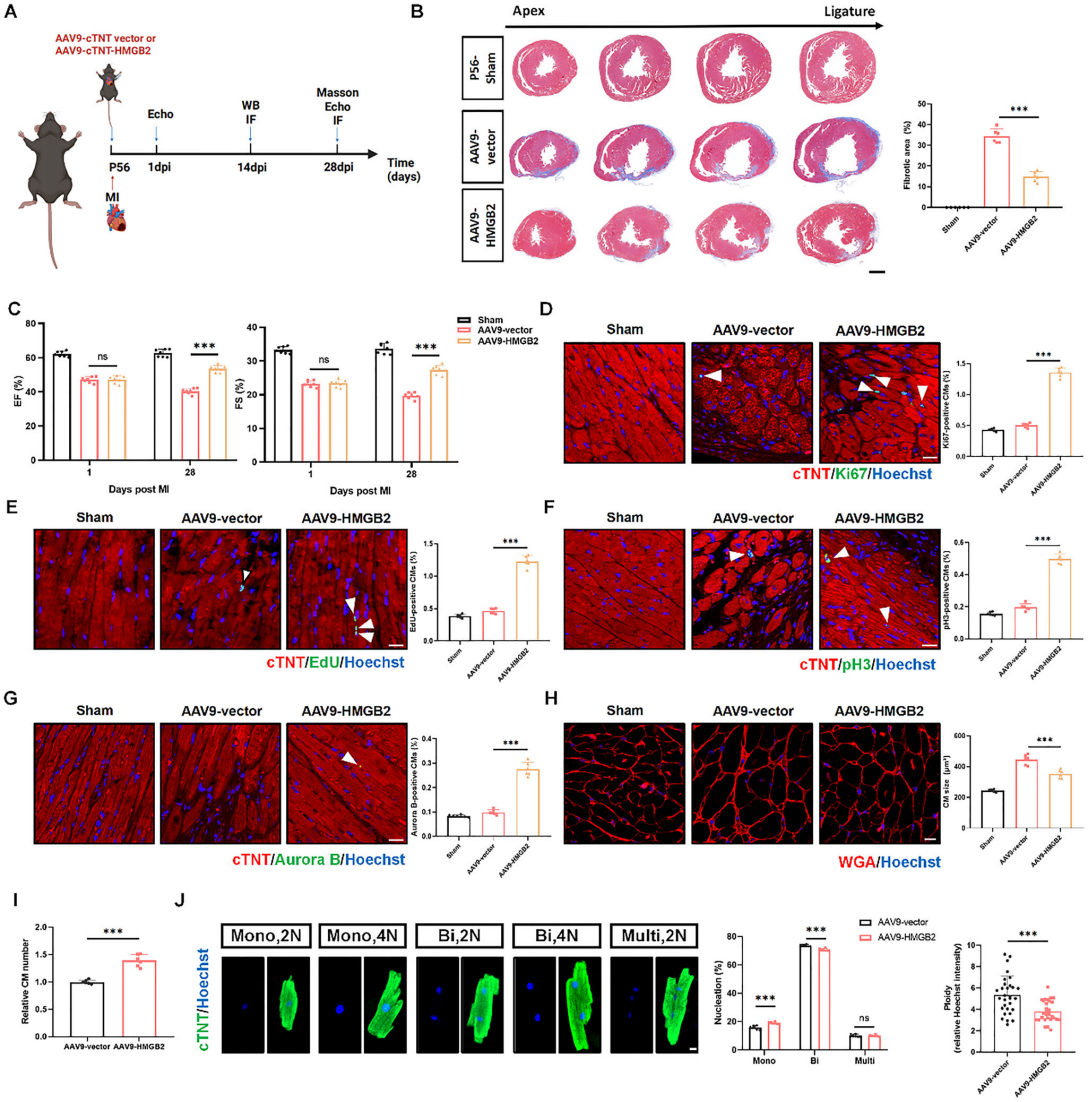
HMGB2 overexpression promotes cardiac regeneration and repair in adult mice after myocardial infarction. A, Schematic representation of the injection of AAV9‐cTNT vector or AAV9‐cTNT‐HMGB2 into the infarct border zone of adult mice after MI (Created in https://BioRender.com). B, Representative Masson's trichrome staining and quantification of fibrotic area in sham mice and MI mice injected with AAV9‐cTNT vector or AAV9‐cTNT‐HMGB2 at 28 dpi (n=6 each). scale bar, 1000 µm. C, Echocardiography analysis of the left ventricle ejection fraction (EF) and fractional shortening (FS) in sham mice and MI mice injected with AAV9‐cTNT vector or AAV9‐cTNT‐HMGB2 at 1 dpi and 28 dpi (n=7 each). D, Ki67 immunofluorescence staining and quantification of Ki67‐positive CMs in sham mice and MI mice injected with AAV9‐cTNT vector or AAV9‐cTNT‐HMGB2 at 14 dpi (n=6 each). cTNT (red), Ki67 (green), Hoechst (blue). scale bar, 20 µm. E, EdU immunofluorescence staining and quantification of EdU‐positive CMs in sham mice and MI mice injected with AAV9‐cTNT vector or AAV9‐cTNT‐HMGB2 at 14 dpi (n=6 each). cTNT (red), EdU (green), Hoechst (blue). scale bar, 20 µm. F, pH3 immunofluorescence staining and quantification of pH3‐positive CMs in sham mice and MI mice injected with AAV9‐cTNT vector or AAV9‐cTNT‐HMGB2 at 14 dpi (n=6 each). cTNT (red), pH3 (green), Hoechst (blue). scale bar, 20 µm. G, Aurora B immunofluorescence staining and quantification of Aurora B‐positive CMs in sham mice and MI mice injected with AAV9‐cTNT vector or AAV9‐cTNT‐HMGB2 at 14 dpi (n=6 each). cTNT (red), Aurora B (green), Hoechst (blue). scale bar, 20 µm. H, WGA immunofluorescence staining and quantification of CM size in sham mice and MI mice injected with AAV9‐cTNT vector or AAV9‐cTNT‐HMGB2 at 28 dpi (n=6 each). WGA (red), Hoechst (blue). scale bar, 10 µm. I, Quantification of CM number in CMs isolated from MI mice injected with AAV9‐cTNT vector or AAV9‐cTNT‐HMGB2 at 28 dpi (n=6 each). J, Representative images and quantification of CM nucleation and ploidy in CMs isolated from MI mice injected with AAV9‐cTNT vector or AAV9‐cTNT‐HMGB2 at 28 dpi (n=6 each). The percentage of mononucleated (mono), binucleated (bi), and multinucleated (multi) CMs in total CMs. cTNT (green), Hoechst (blue). scale bar, 10 µm. Statistical analysis was performed by an unpaired Student's *t*‐test for I‐J. Statistical analysis was performed by one‐way ANOVA followed by Tukey multiple comparisons for B, D‐H. Statistical analysis was performed by two‐way ANOVA followed by Tukey multiple comparisons for C. Ns, not significant. ^***^ *P*<0.001.

To assess whether HMGB2 overexpression could improve cardiac regeneration and repair in adult mice (56‐day‐old mice) following injury, we established an MI model in adult mice and injected AAV9‐cTNT‐HMGB2 or AAV9‐cTNT vector into the infarct border zone (**Figure** **4**A). Western blot and qRT‐PCR analysis confirmed increased HMGB2 mRNA and protein levels in the myocardium of mice injected with AAV9‐cTNT‐HMGB2 (Figure S9A–B, Supporting Information). Masson's trichrome staining and echocardiography showed that HMGB2 overexpression significantly reduced infarct size and improved cardiac function compared to the AAV9‐cTNT vector group (Figure 4B,C). IF staining for proliferation indicators (Ki67, EdU, pH3, and Aurora B) demonstrated a significant increase in the proportion of Ki67, EdU, pH3, and Aurora B‐positive cardiomyocytes following HMGB2 overexpression compared with mice treated with AAV9‐cTNT vector (Figure 4D–G). WGA staining showed that the cross‐sectional area of cardiomyocytes was smaller in the HMGB2 overexpression group at 28 days post‐infarction (dpi) (Figure 4H). Furthermore, IF staining of cardiomyocytes isolated from the HMGB2 and vector groups showed an increase in total cardiomyocyte number and mononucleated cardiomyocytes with decreased ploidy in the HMGB2 group compared to the vector group at 28 dpi (Figure 4I,J). Collectively, these results suggest that HMGB2 protects against myocardial injury by stimulating cardiomyocytes to re‐enter the cell cycle, indicating that AAV9‐mediated gene therapy may be a practical approach to upregulate HMGB2 and promote cardiac regeneration and repair following myocardial injury.

### 2.6 HMGB2 Regulates Glucose Metabolism to Stimulate Cardiomyocyte Proliferation Through the HIF‐1α Pathway

**Figure 5:**
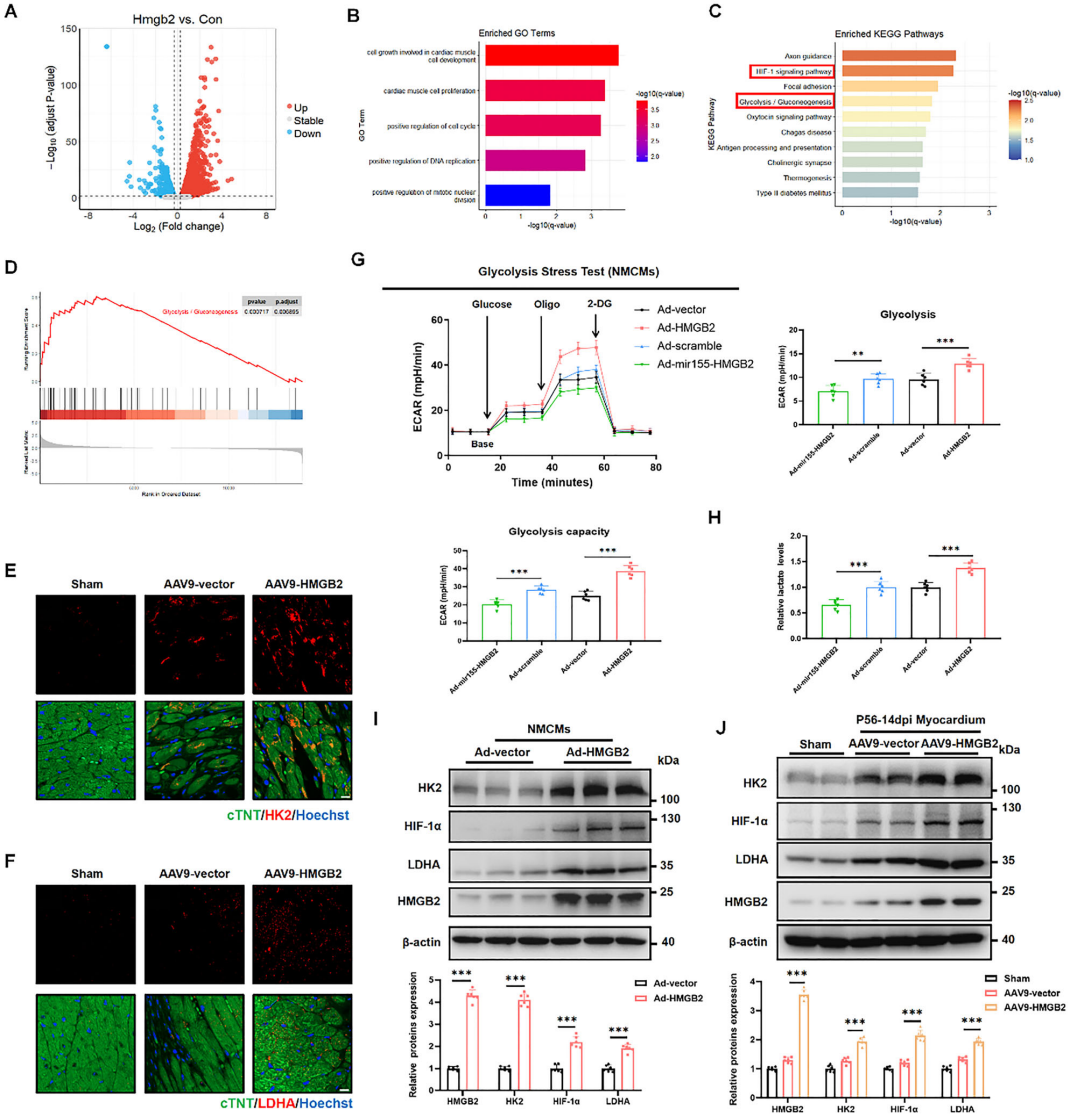
HMGB2 stimulates cardiomyocyte proliferation through HIF‐1α‐mediated glycolysis. A, Volcano plots of differentially expressed genes in NMCMs from the HMGB2 group compared with the vector group (adjusted P value < 0.05 and fold change > 1.2 or < 1/1.2). Red represents upregulated genes and blue represents downregulated genes (n=3 each). B, Gene Ontology (GO) term analysis of differentially expressed genes in cardiomyocytes from HMGB2 group and the vector group. C, Kyoto Encyclopedia of Genes and Genomes (KEGG) term analysis of the enriched signaling pathways in cardiomyocytes from HMGB2 group and vector group. D, Gene Set Enrichment Analysis (GSEA) analysis of gene clusters in the regulation of glycolysis /gluconeogenesis. E, Immunofluorescence staining of HK2 expression in sham mice and MI mice injected with AAV9‐cTNT vector or AAV9‐cTNT‐HMGB2 at 14 dpi. cTNT (green), HK2 (red), Hoechst (blue). scale bar, 10 µm. F, Immunofluorescence staining of LDHA expression in sham mice and MI mice injected with AAV9‐cTNT vector or AAV9‐cTNT‐HMGB2 at 14 dpi. cTNT (green), LDHA (red), Hoechst (blue). scale bar, 10 µm. G, Seahorse XF analysis of glycolysis stress tests in NMCMs transfected with Ad5‐cTNT‐mir155‐HMGB2, Ad5‐cTNT‐mir155‐scramble, Ad5‐cTNT vector, or Ad5‐cTNT‐HMGB2 for 24 h (n=6 each). H, Detection of intracellular lactate concentration in NMCMs transfected with Ad5‐cTNT‐mir155‐HMGB2, Ad5‐cTNT‐mir155‐scramble, Ad5‐cTNT vector, or Ad5‐cTNT‐HMGB2 for 24 h (n=6 each). I, Western blot and quantification of HMGB2, LDHA, HK2, and HIF‐1α proteins expression in NMCMs transfected with Ad5‐cTNT vector or Ad5‐cTNT‐HMGB2 for 24 h (n=6 each). J, Western blot and quantification of HMGB2, LDHA, HK2, and HIF‐1α proteins expression in sham mice and MI mice injected with AAV9‐cTNT vector or AAV9‐cTNT‐HMGB2 at 14 dpi (n=6 each). Statistical analysis was performed by an unpaired Student's *t*‐test for G‐I. Statistical analysis was performed by one‐way ANOVA followed by Tukey multiple comparisons for J. ^**^ *P*<0.01; ^***^ *P*<0.001.

To investigate the downstream signaling pathways by which HMGB2 promotes cardiomyocyte proliferation, RNA‐seq was performed on NMCMs transfected with Ad5‐cTNT‐HMGB2 or Ad5‐cTNT vector. Compared to the vector group, 2,365 genes were upregulated, and 1,702 genes were downregulated in the HMGB2 group (**Figure** **5**A; Figure S10A, Supporting Information). Gene ontology (GO) enrichment analysis revealed that differentially expressed genes were mainly involved in processes such as cardiomyocyte growth and cardiomyocyte proliferation (Figure 5B). Kyoto Encyclopedia of Genes and Genomes (KEGG) enrichment analysis revealed significant enrichment in the HIF‐1 signaling pathway and Glycolysis/Gluconeogenesis following HMGB2 overexpression (Figure 5C). Additionally, gene set enrichment analysis (GSEA) showed that HMGB2 overexpression resulted in a dynamic bias toward positive regulation of glycolysis, a process closely associated with cardiomyocyte proliferation and heart regeneration^[^ ^19^, ^20^, ^21^, ^22^, ^23^ ^]^ (Figure 5D). Based on these results, we speculated that HMGB2 might affect cardiomyocyte proliferation through regulation of glycolysis. Subsequently, qRT‐PCR validation of genes related to the glycolysis/gluconeogenesis pathway (Eno2, HK2, LDHA, PGK1, Eno3, and PFKL) demonstrated that HMGB2 overexpression significantly increased the mRNA levels of these genes, whereas HMGB2 knockdown decreased their expression (Figure S10B, Supporting Information). IF staining further confirmed that HMGB2 overexpression enhanced the HK2 and LDHA expression in the myocardium of adult mice following MI (Figure 5E,F). To assess the impact of HMGB2 on glycolysis in NMCMs, extracellular acidification rate (ECAR) was measured, indicating that HMGB2 overexpression enhanced glycolysis capacity and increased lactate production, whereas HMGB2 knockdown had the opposite effect (Figure 5G,H). Collectively, these findings indicate that HMGB2 is crucial for cardiomyocyte proliferation through regulation of glycolysis.

As a key factor in the HIF‐1 signaling pathway, the transcriptional activator HIF‐1α is closely related to glucose metabolism and is positively correlated with the expression of glycolysis‐related genes.^[^ ^24^, ^25^, ^26^, ^27^, ^28^ ^]^ We therefore hypothesized that HMGB2 might promote cardiomyocyte proliferation through HIF‐1α‐mediated glycolysis. To test this hypothesis, the expression levels of HIF‐1α and glycolysis‐related proteins in cardiomyocytes with either HMGB2 overexpression or knockdown were examined. Significantly, the expression levels of HIF‐1α and glycolysis‐related proteins (HK2 and LDHA) were positively correlated with HMGB2 expression (Figure 5I,J; Figure S10C,D, Supporting Information). To further investigate the role of HIF‐1α in heart regeneration, its expression was analyzed at 1 and 3 dpr in P1 mice, where it was found to be significantly upregulated at both time points (Figure S11A, Supporting Information). Following this, the effect of HIF‐1α on cardiomyocyte proliferation was explored through gene therapy (Figure S11B, Supporting Information). Our results showed that HIF‐1α overexpression not only upregulated glycolysis‐related proteins (HK2 and LDHA) but also increased lactate levels (Figure S11C–F, Supporting Information). Moreover, HIF‐1α overexpression increased the proportion of Ki67, EdU, and pH3‐positive cardiomyocytes, and reduced the cross‐sectional area of cardiomyocytes (Figure S11G–J, Supporting Information). Thus, we conclude that HMGB2 promotes cardiomyocyte proliferation by activating HIF‐1α‐mediated glycolysis in adult mice following MI.

To further corroborate this conclusion, we performed rescue experiments using the previously reported strategy^[^ ^29^ ^]^ (Figure S12A, Supporting Information). HMGB2 overexpression significantly increased expression levels of glycolysis‐related proteins (HK2 and LDHA), while HIF‐1α knockdown attenuated this effect (Figure S12B, Supporting Information). Furthermore, our results showed that HIF‐1α knockdown significantly reversed the effect of HMGB2 overexpression on activating glycolysis, enhancing cardiomyocyte proliferation, improving cardiac function, and reducing infarct size in adult mice following MI (Figure S12C–I, Supporting Information). These results suggest that HIF‐1α knockdown eliminates the protective effect of HMGB2 overexpression on cardiac regeneration and repair in adult mice after MI.

### 2.7 HMGB2 Interacts with MTA2 Through its Δ80‐165 Region

**Figure 6:**
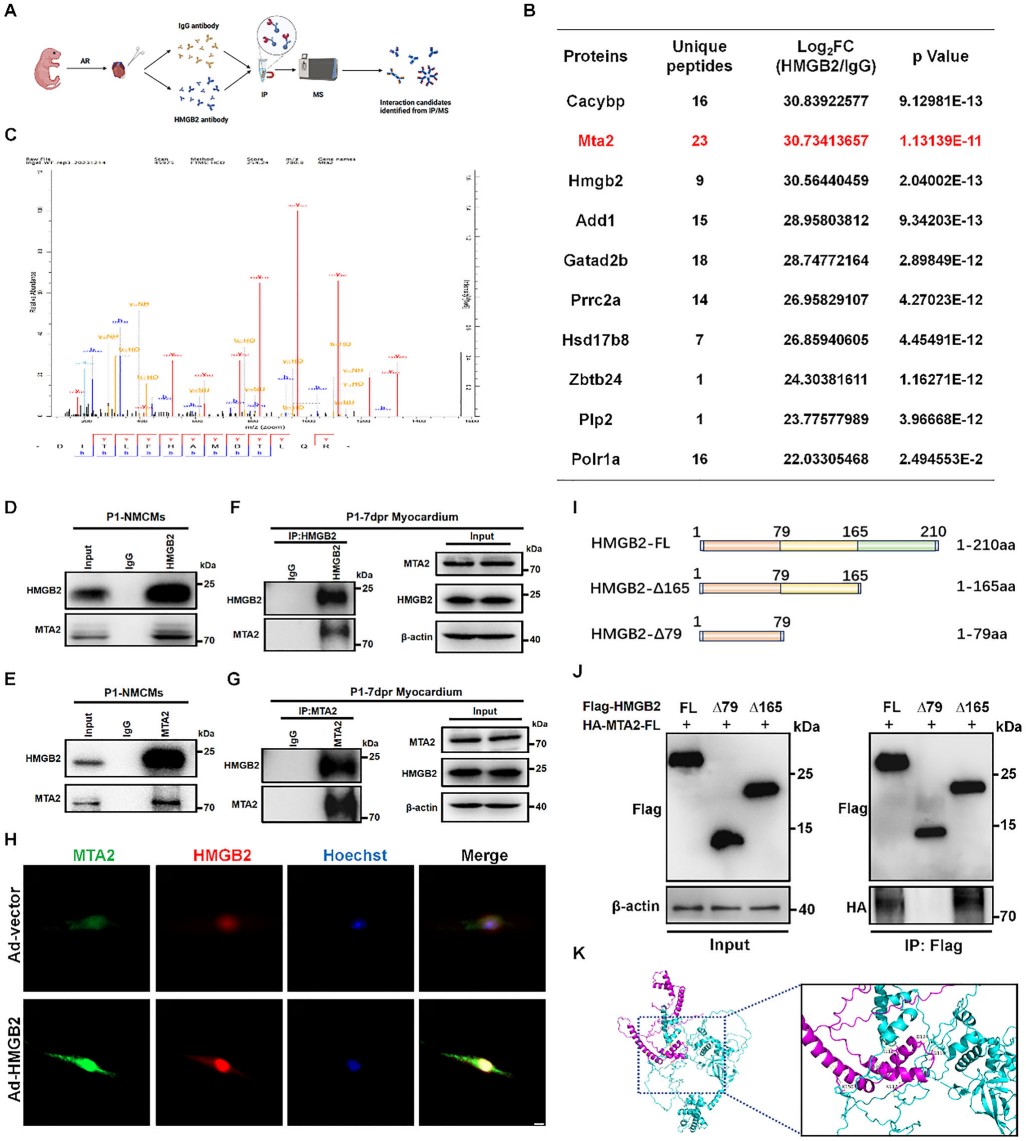
The Δ80‐165 domain of HMGB2 binds to MTA2. A, Schematic diagram of the procedure identifying downstream targets of HMGB2 (Created in https://BioRender.com). B, Identification of MTA2 as the downstream target of HMGB2 by immunoprecipitation coupled with mass spectrometry (IP/MS) analysis. C, Identification of MTA2 as a possible protein that interacts with HMGB2. D‐E, CO‐immunoprecipitation (CO‐IP) and Western blot analysis of the interaction of HMGB2 and MTA2 in NMCMs. F‐G, CO‐IP and Western blot analysis of HMGB2 and MTA2 protein interactions in neonatal mouse myocardium at 7 dpr. H, Immunofluorescence staining analysis of HMGB2 and MTA2 protein interactions in NMCMs transfected with Ad5‐cTNT vector or Ad5‐cTNT‐HMGB2 for 24 h. HMGB2 (red), MTA2 (green), Hoechst (blue). scale bar, 5 µm. I, Design diagram of HMGB2 full‐length and truncated plasmids (∆1‐165 and ∆1‐79). J, CO‐IP and Western blot analysis of Flag‐HMGB2 and HA‐MTA2 interaction in HEK293T cells transfected with HA‐MTA2‐FL and truncated Flag‐HMGB2 plasmids in I. K, Molecular docking analysis of the interaction between the MTA2 and HMGB2 domains, with HMGB2 in magenta and MTA2 in cyan.

To evaluate the downstream targets of HMGB2 in activating HIF‐1α‐mediated glycolysis and promoting heart regeneration, immunoprecipitation coupled with mass spectrometry (IP‐MS) was conducted to identify proteins interacting with HMGB2 in the myocardium of P1 mice following AR (**Figure** **6**A). A total of 94 proteins were identified, including MTA2, which was shown to be involved in the stabilization of HIF‐1α through deacetylation.^[^ ^30^ ^]^ Next, MTA2 was selected as a potential downstream target of HMGB2 (Figure 6B,C). In vitro, co‐immunoprecipitation (CO‐IP) confirmed the interaction between HMGB2 and MTA2 in NMCMs (Figure 6D,E). We further verified that HMGB2 interacted with MTA2 through CO‐IP assays using HMGB2 or MTA2 antibodies in P1 mouse myocardium at 7 dpr (Figure 6F,G). Notably, IF staining showed that HMGB2 overexpression promoted the binding of HMGB2 and MTA2 in the nucleus (Figure 6H). To pinpoint the interaction domains of HMGB2 and MTA2, Flag‐tagged HMGB2 truncated plasmids Δ165 and Δ79 were constructed based on the UniProt database^[^ ^11^ ^]^ (Figure 6I). These truncated plasmids were transfected into HEK293T cell lines, and further CO‐IP analysis revealed that HMGB2Δ165 and full‐length HMGB2 could immunoprecipitate with HA‐MTA2, whereas HMGB2Δ79 could not bind to HA‐MTA2 (Figure 6J). These findings were consistent with the interaction model predicted by the ClusPro server (Figure 6K). These results indicate that amino acids 80‐165 of HMGB2 mediate its interaction with MTA2.

### 2.8 HMGB2 Inhibits the Ubiquitination Degradation of MTA2 to Stabilize HIF‐1α

**Figure 7:**
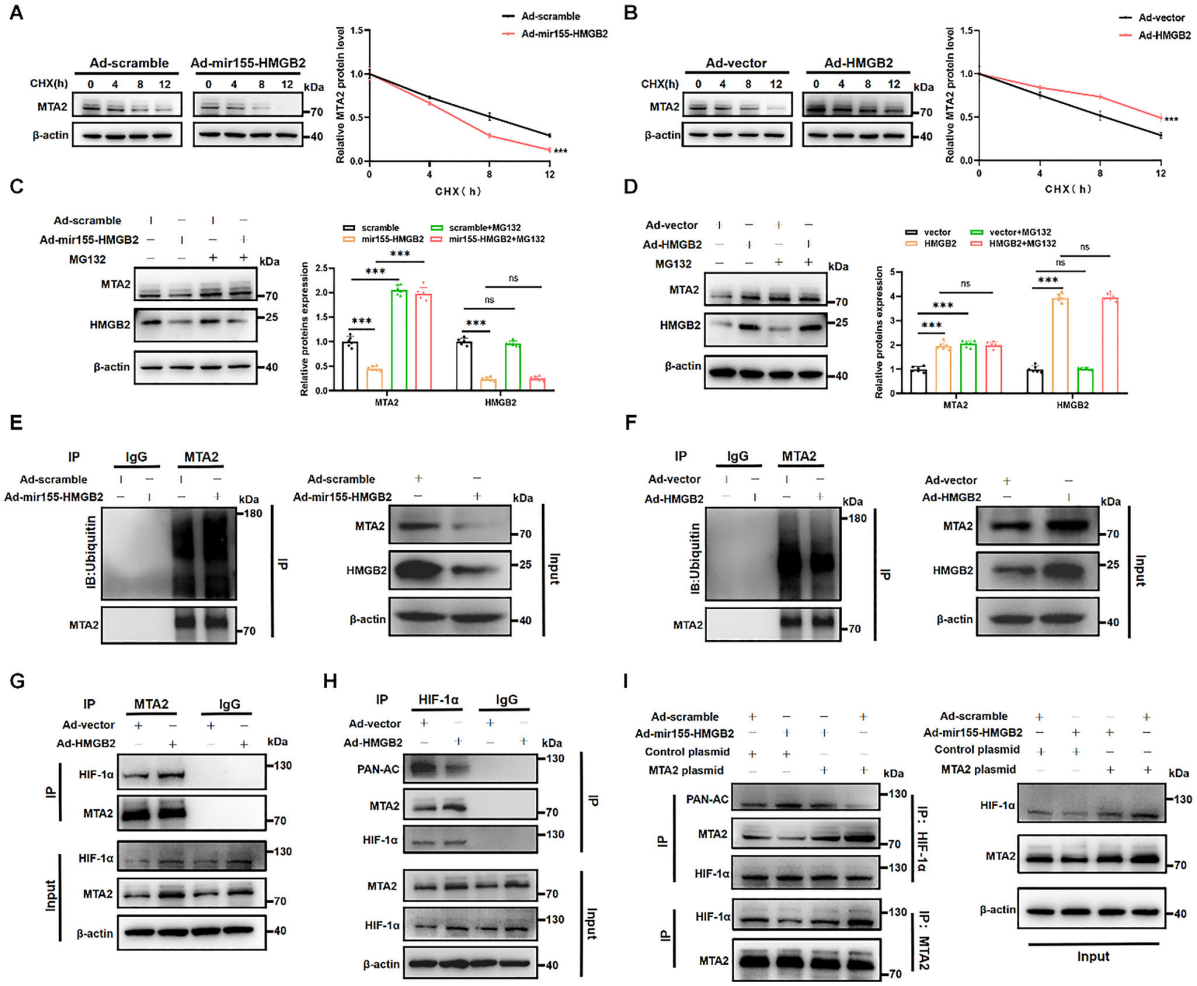
HMGB2 inhibits MTA2 degradation through the ubiquitin‐proteasome pathway and stabilizes HIF‐1α level through the deacetylation pathway. A, Western blot and quantification of MTA2 protein stability in NMCMs transfected with Ad5‐cTNT‐mir155‐scramble or Ad5‐cTNT‐mir155‐HMGB2 after treating with CHX (200 µM) for 0, 4, 8, 12h (n=6 each). CHX, cycloheximide. B, Western blot and quantification of MTA2 protein stability in NMCMs transfected with Ad5‐cTNT vector or Ad5‐cTNT‐HMGB2 after treating with CHX (200 µM) for 0, 4, 8, 12h (n=6 each). C, Western blot and quantification of MTA2 protein expression in NMCMs transfected with Ad5‐cTNT‐mir155‐scramble or Ad5‐cTNT‐mir155‐HMGB2 after treating with or without MG132 (20 µM). D, Western blot and quantification of MTA2 protein expression in NMCMs transfected with Ad5‐cTNT vector or Ad5‐cTNT‐HMGB2 after treating with or without MG132 (20 µM). E, CO‐IP and Western blot analysis of the ubiquitination level of MTA2 in NMCMs transfected with Ad5‐cTNT‐mir155‐scramble or Ad5‐cTNT‐mir155‐HMGB2. F, CO‐IP, and Western blot analysis of the ubiquitination level of MTA2 in NMCMs transfected with Ad5‐cTNT vector or Ad5‐cTNT‐HMGB2. G, CO‐IP, and Western blot analysis of HIF‐1α and MTA2 protein interactions in NMCMs transfected with Ad5‐cTNT vector or Ad5‐cTNT‐HMGB2. H, CO‐IP, and Western blot analysis of the acetylation level of HIF‐1α and the interaction between MTA2 and HIF‐1α proteins in NMCMs transfected with Ad5‐cTNT vector or Ad5‐cTNT‐HMGB2. I, CO‐IP and Western blot analysis of the acetylation of HIF‐1α and the interaction between MTA2 and HIF‐1α proteins in NMCMs transfected with Ad5‐cTNT‐mir155‐scramble or Ad5‐cTNT‐mir155‐HMGB2 and control plasmid or MTA2 plasmid. Statistical analysis was performed by an unpaired Student's *t*‐test for A‐B. Statistical analysis was performed by two‐way ANOVA followed by Tukey multiple comparisons for C‐D. Ns, not significant. ^***^ *P*<0.001.

Next, the regulation of MTA2 expression by HMGB2 was explored. Western blot analysis demonstrated that MTA2 protein expression was significantly increased with HMGB2 overexpression and notably decreased with HMGB2 knockdown, while MTA2 mRNA expression levels remained unchanged regardless of HMGB2 expression (Figure S13A–C, Supporting Information). To investigate the mechanism by which HMGB2 regulates MTA2, NMCMs were treated with adenoviruses targeting HMGB2 and the protein synthesis inhibitor cycloheximide (CHX) to track MTA2 degradation over time. Western blot results demonstrated that HMGB2 stabilized MTA2 in the presence of CHX (**Figure** **7**A,B). Furthermore, treatment with the proteasome inhibitor MG132 reversed the reduction in MTA2 protein expression level induced by HMGB2 knockdown, indicating that HMGB2 maintains MTA2 protein stability via the proteasome pathway (Figure 7C,D). Further CO‐IP of MTA2 revealed an increase in MTA2 ubiquitination with HMGB2 knockdown, while MTA2 ubiquitination decreased with HMGB2 overexpression (Figure 7E,F). In summary, our findings suggest that HMGB2 binds to MTA2 in cardiomyocytes and inhibits its degradation in a ubiquitin‐proteasome dependent manner.

Prior research has shown that MTA2 stabilizes HIF‐1α via promoting its deacetylation,^[^ ^30^, ^31^ ^]^ yet its function in cardiomyocytes remains to be clarified. Thus, we aimed to determine whether HMGB2 enhances the stabilization of HIF‐1α by modulating MTA2 in NMCMs. CO‐IP with antibodies against MTA2 or HIF‐1α demonstrated an enhanced interaction and a reduction in the acetylation of HIF‐1α in NMCMs transfected with Ad5‐cTNT‐HMGB2 (Figure 7G,H). Furthermore, overexpression of MTA2 was found to restore both the interaction and inhibit the acetylation of HIF‐1α following HMGB2 knockdown (Figure 7I). These findings imply that HMGB2 overexpression stabilizes HIF‐1α by enhancing MTA2 expression.

### 2.9 HMGB2 Promotes Cardiomyocyte Proliferation and Cardiac Repair Through the MTA2/HIF‐1α Signaling Pathway

Investigation into the role of MTA2 in heart regeneration revealed a significant increase in MTA2 protein expression in the myocardium of P1 mice at 1 and 3 dpr (Figure S14A, Supporting Information). An MTA2 plasmid was then constructed to assess its impact on cardiomyocyte proliferation in vitro, with Western blot and qRT‐PCR confirming successful MTA2 transfection (Figure S14B–C, Supporting Information). Following MTA2 overexpression, there was a notable enhancement in glycolysis, along with increased expression levels of HIF‐1α and glycolysis‐related proteins (HK2 and LDHA), as well as elevated lactate production (Figure S14D–F, Supporting Information). Moreover, an increase in cardiomyocyte number and the proportion of EdU‐positive cardiomyocytes, Ki67‐positive cardiomyocytes, as well as pH3‐positive cardiomyocytes was observed after MTA2 overexpression (FigureS14G–J, Supporting Information). Taken together, these results suggest that MTA2 drives cardiomyocyte proliferation by facilitating HIF‐1α‐mediated glycolysis.

To assess whether the MTA2/HIF‐1α axis‐mediated glycolysis contributes to HMGB2's regulation of cardiomyocyte proliferation, NMCMs were transfected with Ad5‐cTNT‐mir155‐HMGB2 or Ad5‐cTNT‐mir155‐scramble and MTA2 plasmid or control plasmid. We observed that HMGB2 knockdown significantly reduced the expression levels of HIF‐1α and glycolysis‐associated proteins (HK2 and LDHA), whereas MTA2 overexpression mitigated this effect (Figure S15A, Supporting Information). Additionally, MTA2 overexpression considerably restored glycolysis capacity and elevated lactate production in NMCMs transfected with Ad5‐cTNT‐mir155‐HMGB2 (Figure S15B,C, Supporting Information). We performed function‐rescue experiments and demonstrated that MTA2 overexpression counteracted the adverse effects of HMGB2 knockdown on cardiomyocyte proliferation (Figure S15D–H, Supporting Information).

To further elucidate the interaction between HMGB2 and MTA2 in regulating heart regeneration in vivo, we conducted rescue experiments in neonatal mice following AR. Western blot and lactate detection revealed that MTA2 knockdown decreased the expression levels of HIF‐1α and glycolysis‐related proteins (HK2 and LDHA), along with lactate production, effects which were reversed by HMGB2 overexpression in the neonatal AR model (Figure S16A,B, Supporting Information). Moreover, HMGB2 overexpression abrogated the decreased proliferative rate and mitigated the increase in cardiomyocyte cross‐sectional area induced by MTA2 knockdown in the neonatal AR model (Figure S16C–G, Supporting Information).

**Figure 8:**
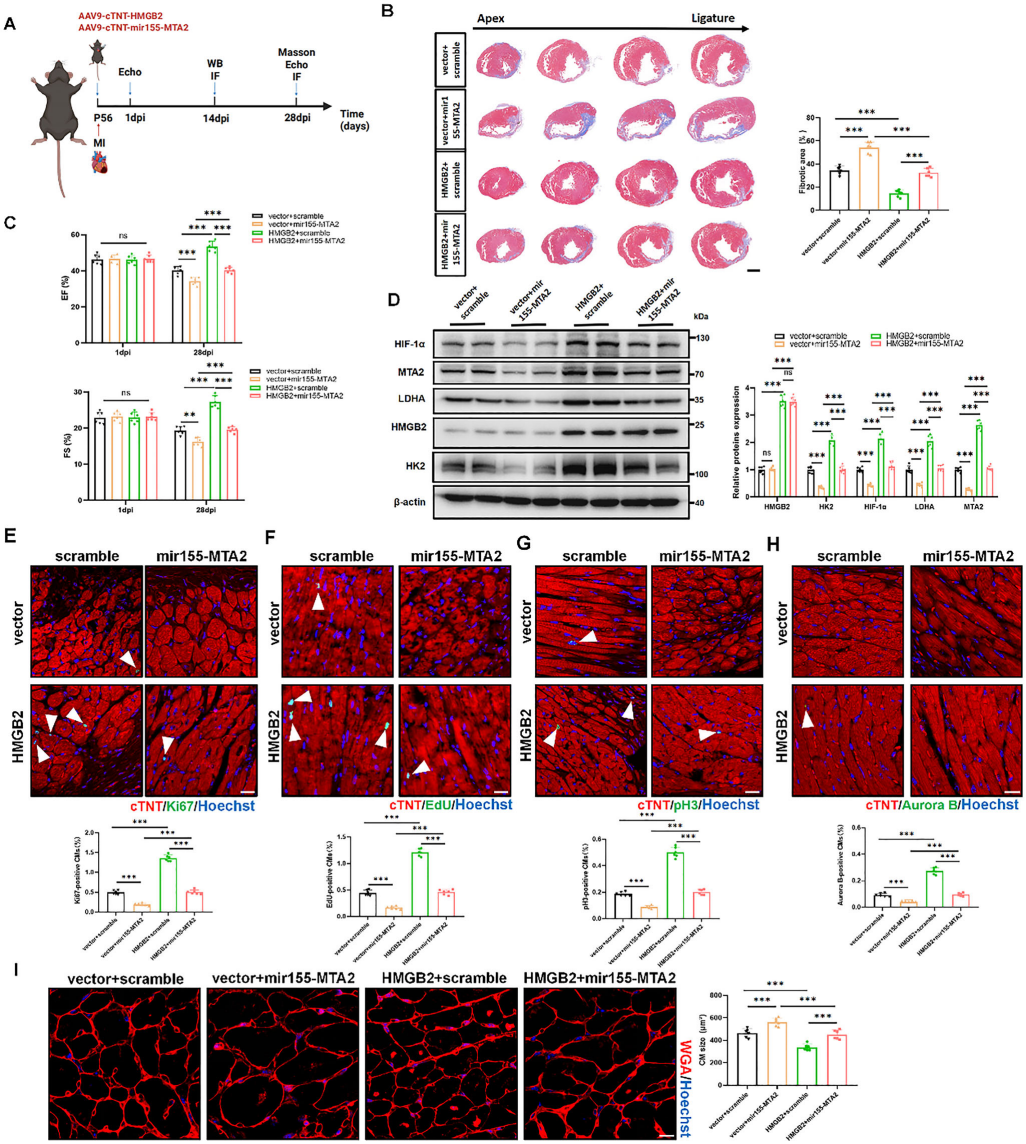
MTA2 knockdown diminishes the protective effects of HMGB2 on cardiac regeneration and repair in adult mice after myocardial infarction. A, Schematic representation of the injection of AAV9‐cTNT‐HMGB2 or AAV9‐cTNT vector and AAV9‐cTNT‐mir155‐scramble or AAV9‐cTNT‐mir155‐MTA2 into the infarct border zone of adult mice after myocardial infarction (Created in https://BioRender.com). B, Representative Masson's trichrome staining and quantification of fibrotic area in MI mice injected with AAV9‐cTNT vector or AAV9‐cTNT‐HMGB2 and AAV9‐cTNT‐mir155‐scramble or AAV9‐cTNT‐mir155‐MTA2 at 28 dpi (n=6 each). scale bar, 1000 µm. C, Echocardiography analysis of the left ventricle ejection fraction (EF) and fractional shortening (FS) in MI mice injected with AAV9‐cTNT vector or AAV9‐cTNT‐HMGB2 and AAV9‐cTNT‐mir155‐scramble or AAV9‐cTNT‐mir155‐MTA2 at 1 dpi and 28 dpi (n=6 each). D, Western blot and quantification of HMGB2, MTA2, LDHA, HK2, and HIF‐1α proteins expression in MI mice injected with AAV9‐cTNT vector or AAV9‐cTNT‐HMGB2 and AAV9‐cTNT‐mir155‐scramble or AAV9‐cTNT‐mir155‐MTA2 at 14 dpi (n=6 each). E, Ki67 immunofluorescence staining and quantification of Ki67‐positive CMs in MI mice injected with AAV9‐cTNT vector or AAV9‐cTNT‐HMGB2 and AAV9‐cTNT‐mir155‐scramble or AAV9‐cTNT‐mir155‐MTA2 at 14 dpi (n=6 each). cTNT (red), Ki67 (green), Hoechst (blue). scale bar, 20 µm. F, EdU immunofluorescence staining and quantification of EdU‐positive CMs in MI mice injected with AAV9‐cTNT vector or AAV9‐cTNT‐HMGB2 and AAV9‐cTNT‐mir155‐scramble or AAV9‐cTNT‐mir155‐MTA2 at 14 dpi (n=6 each). cTNT (red), EdU (green), Hoechst (blue). scale bar, 20 µm. G, pH3 immunofluorescence staining and quantification of pH3‐positive CMs in MI mice injected with AAV9‐cTNT vector or AAV9‐cTNT‐HMGB2 and AAV9‐cTNT‐mir155‐scramble or AAV9‐cTNT‐mir155‐MTA2 at 14 dpi (n=6 each). cTNT (red), pH3 (green), Hoechst (blue). scale bar, 20 µm. H, Aurora B immunofluorescence staining and quantification of Aurora B‐positive CMs in MI mice injected with AAV9‐cTNT vector or AAV9‐cTNT‐HMGB2 and AAV9‐cTNT‐mir155‐scramble or AAV9‐cTNT‐mir155‐MTA2 at 14 dpi (n=6 each). cTNT (red), Aurora B (green), Hoechst (blue). scale bar, 20 µm. I, WGA immunofluorescence staining and quantification of CM size in MI mice injected with AAV9‐cTNT vector or AAV9‐cTNT‐HMGB2 and AAV9‐cTNT‐mir155‐scramble or AAV9‐cTNT‐mir155‐MTA2 at 28 dpi (n=6 each). WGA (red), Hoechst (blue). scale bar, 10 µm. Statistical analysis was performed by two‐way ANOVA followed by Tukey multiple comparisons for B‐I. Ns, not significant. ^**^ *P*<0.01; ^***^ *P*<0.001.

Next, we examined whether MTA2 knockdown in adult mice would affect HMGB2's role in promoting cardiac regeneration and repair following MI (**Figure** **8**A). Echocardiography and Masson's trichrome staining results revealed that MTA2 knockdown significantly eliminated the protective effects of HMGB2 overexpression in improving cardiac function and reducing infarct size (Figure 8B,C). Remarkably, increased expression levels of HIF‐1α and glycolysis‐related proteins (HK2 and LDHA) were noted in the myocardium of mice injected with AAV9‐cTNT‐HMGB2, whereas co‐injection with AAV9‐cTNT‐HMGB2 and AAV9‐cTNT‐mir155‐MTA2 reversed these enhancements (Figure 8D). A decrease in the production of lactate was also observed in cardiomyocytes from mice co‐injected with AAV9‐cTNT‐HMGB2 and AAV9‐cTNT‐mir155‐MTA2 compared to those injected only with AAV9‐cTNT‐HMGB2 (Figure S17A, Supporting Information). Further analysis revealed that MTA2 knockdown blocked the favorable effects of HMGB2 overexpression on cardiomyocyte proliferation and prevented reductions in cardiomyocyte cross‐sectional area subsequent to HMGB2 overexpression (Figure 8E–I). In conclusion, both in vitro and in vivo data indicate that MTA2, as a downstream mediator of HMGB2, is crucial for fostering cardiac regeneration and repair in mice following myocardial injury.

## 3 Discussion

In this study, we employed a combination of quantitative proteomics with tandem mass tag labeling and RNA‐seq analysis and uncovered novel insights into the role of HMGB2 in heart regeneration. We found that the changes in both protein and mRNA expression levels of HMGB2 were consistent with the patterns observed in heart regeneration. In addition, HMGB2 was crucial for extending the regenerative window of cardiomyocytes and promoting heart regeneration. Moreover, HMGB2 facilitated cardiomyocyte proliferation via regulating metabolic reprogramming. Further, the Δ80‐165 region of HMGB2 interacted with MTA2 and prevented its degradation through the ubiquitin‐proteasome pathway, thereby activating HIF‐1α‐mediated glycolysis by inhibiting HIF‐1α acetylation (Figure S18, Supporting Information). These findings together reveal a novel role of HMGB2 in promoting heart regeneration for the first time.

HMGB2, a high‐mobility group domain protein, predominantly localizes in the nucleus and plays a vital role in cell proliferation and differentiation.^[^ ^10^, ^11^, ^32^ ^]^ Previous studies have demonstrated that knockdown of HMGB2 impairs cardiac function and increases susceptibility to heart failure after transverse aortic constriction.^[^ ^18^ ^]^ However, the biological function of HMGB2 in heart regeneration remains unexplored. Our findings revealed that HMGB2 expression in the myocardium decreased with age during postnatal development. We demonstrated that HMGB2 overexpression was essential for cardiomyocyte proliferation and cardiac repair in both neonatal and adult mice after myocardial injury, as shown through a series of comprehensive experimental models. AAV9 was used in this study instead of lentivirus due to its high transduction efficiency in cardiomyocytes, uniform myocardial distribution, and strong specificity for cardiomyocytes.^[^ ^29^, ^33^, ^34^, ^35^, ^36^, ^37^, ^38^, ^39^, ^40^, ^41^ ^]^ Importantly, our results have shown that the delivery of AAV9‐cTNT‐HMGB2 effectively stimulates cardiomyocytes to re‐enter the cell cycle, offering a promising approach for AAV9‐mediated gene therapy in heart regeneration following myocardial injury. These findings underscore the potential of HMGB2 as a therapeutic target for heart regeneration, thereby advancing our understanding of the molecular network mechanisms that activate regenerative programs after injury.

The neonatal mammalian heart exhibits a significant yet transient capacity for regeneration following injury, a capability that notably declines after the first week post‐birth.^[^ ^42^, ^43^, ^44^, ^45^ ^]^ A pivotal factor contributing to the diminishing regenerative potential is a transformation in cardiomyocyte energy metabolism, transitioning from glycolysis to fatty acid oxidation as the primary energy source during postnatal development.^[^ ^22^, ^29^, ^46^, ^47^ ^]^ Previous findings have indicated that as neonatal cardiomyocytes mature, there is a noticeable decrease in the expression levels of critical glycolytic enzymes, resulting in a marked decline in glycolysis capacity from P2 to P7.^[^ ^19^, ^48^, ^49^ ^]^ Thus, the switch to glucose metabolism can initially be beneficial for cardiomyocyte proliferation and heart regeneration.^[^ ^50^, ^51^ ^]^ Our study showed that HMGB2 could promote glycolysis through regulating the HIF‐1α signaling pathway in cardiomyocytes. Based on these results, we further probed the combined regulatory effects of HMGB2 and HIF‐1α on glycolysis and cardiomyocyte proliferation. We found that knockdown of HIF‐1α abolished the protective effects of HMGB2 in promoting cardiomyocyte proliferation and cardiac repair through activating glycolysis in adult mice after myocardial injury, which was consistent with previous studies reporting the transcription factor HIF‐1α in regulating glycolysis to promote cell proliferation and survival.^[^ ^52^, ^53^, ^54^ ^]^ Collectively, our study provides novel evidence indicating that HMGB2 regulates metabolic rewiring to promote cardiomyocyte proliferation through activating the HIF‐1α signaling pathway. To the best of our knowledge, this is the first study to decipher the regulatory impact of HMGB2 in the metabolic reprogramming activity of cardiomyocytes during heart regeneration following myocardial injury.

To investigate the mechanism through which HMGB2 enhances glycolysis via HIF‐1α activation in the myocardium, we employed IP/MS and CO‐IP analyses to identify potential downstream regulators of HMGB2. We identified MTA2 as a potential downstream target of HMGB2. As an integral component of the nucleosome remodeling and deacetylating (NuRD) complex, MTA2 is crucial in gene expression regulation.^[^ ^55^, ^56^, ^57^ ^]^ While prior studies have implicated MTA2 in tumorigenesis, its role in cardiomyocyte proliferation and heart regeneration remains uncertain.^[^ ^56^, ^58^ ^]^ Our study proposes that MTA2 acts as a novel facilitator of cardiomyocyte proliferation and heart regeneration. Notably, previous evidence has shown that MTA2 stabilizes HIF‐1α through deacetylation, thereby promoting cell proliferation.^[^ ^30^, ^31^ ^]^ Our results further demonstrated that HMGB2 modulates MTA2 expression post‐translationally in cardiomyocytes. Specifically, the Δ80‐165 domain of HMGB2 interacts with MTA2 in the nucleus, inhibiting its ubiquitin‐mediated degradation and thus supporting the deacetylation and stabilization of HIF‐1α. Importantly, our experiments also demonstrated that silencing the MTA2/HIF‐1α signaling pathway negates HMGB2's protective effects on cardiomyocyte proliferation and cardiac repair in mice following myocardial injury. These findings highlight the critical role of HMGB2 in enhancing cardiomyocyte proliferation by activating MTA2/HIF‐1α‐dependent glucose metabolism.

The significance and clinical translational potential of the findings in this study are as follows: 1) HMGB2 knockdown inhibits heart regeneration in neonatal mice after AR, while HMGB2 overexpression promotes cardiac regeneration and repair in adult mice following MI, indicating that HMGB2 is a promising target for heart regeneration therapies; 2) HMGB2 drives a metabolic reprogram shift by promoting glycolysis, thus reactivating cardiomyocyte proliferation and supporting heart regeneration; 3) The Δ80‐165 region of HMGB2 binds to and inhibits the ubiquitination degradation of MTA2, highlighting a new mechanistic insight into the role HMGB2 in heart regeneration.

Our study has several limitations: First, though mediated by AAV9, the efficiency of cardiomyocyte‐specific overexpression of HMGB2 is not as high as that seen in transgenic models. A homozygous HMGB2 overexpression mouse model might provide more definitive insights. Second, this study primarily focuses on the role of HMGB2 in cardiomyocytes. However, it is important to note that HMGB2 might have a potential impact on secreted cytokines and other unknown factors, which could have substantial effects on other cell types, such as endothelial cells and fibroblasts. Third, HMGB2 interacts with MTA2 and prevents the ubiquitination of MTA2 through its Δ80–165 region, but the enzymes and specific sites involved in this process require further investigation.

In conclusion, our study elucidates a novel function of the HMGB2‐MTA2‐HIF‐1α axis in regulating glycolysis and promoting cardiomyocyte proliferation. Targeting HMGB2 and its associated pathways holds promise for advancing heart regeneration therapies following injury. The therapeutic implications of this strategy invite further exploration in larger animal models and other cardiovascular conditions.

## 4 Experimental Section

### Animals

All animal experiments in this study were performed in accordance with the Guide for the Use and Care of Laboratory Animals, and the protocols of all animals were approved according to the Institutional Animal Care and Use Committee of Nanjing Medical University (IACUC:1601038). All 1‐day‐old (postnatal day 1; P1) C57BL/6J mice and 56‐day‐old (postnatal day 56; P56) male C57BL/6 mice were sourced from the Animal Core Facility of Nanjing Medical University (Nanjing, China) and were reared in a specific pathogen‐free environment with a 12 h dark/light cycle.

### Apical Resection (AR) Model Construction

AR was performed as previously described.^[^ ^29^, ^59^ ^]^ Briefly, newborn mice at 1 day old (P1) were anesthetized in an ice bath for 3 min. After making an incision in the chest skin using ophthalmic scissors, the fourth intercostal muscle was bluntly dissected with ophthalmic forceps to expose the apex of the heart. Approximately15% of the total left ventricular myocardial tissue was excised using ophthalmic scissors. 6‐0 non‐absorbable sutures were used to suture the chest wall and skin of mice, following which the mice were positioned on a warming stage to facilitate recovery. Once the mice regained consciousness, they were returned to their mothers. A total of 131 P1 mice underwent apical resection surgery in this study. In the sham group, identical procedures were carried out, except that the heart apex was not truncated.

### Myocardial Infarction (MI) Model Construction

The MI model was established using a previously described method.^[^ ^40^ ^]^ Fifty‐six‐day‐old male C57BL/6 mice were selected and anesthetized with an intraperitoneal injection of 1.2% Avertin (200 µL/10g; Sigma–Aldrich, St. Louis, USA). After anesthesia, tracheal intubation was carried out, and the mice were ventilated using a small animal ventilator. The fourth intercostal muscle was gently separated, and MI was induced by applying a 7‐0 non‐absorbable silk suture to ligate the left anterior descending coronary artery (LAD). After the procedure was completed, the incision was closed with 3‐0 non‐absorbable silk sutures. Postoperatively, the mice were placed on a heated platform to facilitate recovery. For the sham operation, the same procedure was performed, but without ligating the LAD coronary artery. The surgeon performing the procedure was blinded to the group assignment and treatment.

### Echocardiography

For echocardiographic measurement, the mice were anesthetized with 0.5–1.0% isoflurane, and assessments were conducted by trained technicians. M‐mode echocardiography was employed to measure left ventricular ejection fraction (LVEF), left ventricular fraction shortening (LVFS), and left ventricular internal diameter (LVID) using a Vevo 2100 or 3100 high‐resolution imaging system (Visual Sonics Inc.). Cardiac function was evaluated at various time points. All echocardiographic evaluations were performed by two experienced technicians who were blinded to the grouping of the mice at the Animal Center of Nanjing Medical University.

### Masson's Trichrome Staining

Mouse hearts were excised and fixed in 4% paraformaldehyde (PFA) at the designated time points, subsequently prepared into 4 µm‐thick heart sections for staining. Masson's Trichrome staining was conducted following a standardized protocol. Images were observed and captured using a microscope. Quantitative analysis of myocardial fibrosis was performed using Image J software, which involved calculating the percentage of fibrotic tissue area (blue) relative to the total left ventricular area (red plus blue). All analyses of Masson's Trichrome staining were conducted in a double‐blinded manner.

### Study Protocols

Adenovirus (Ad5‐cTNT‐mir155‐HMGB2 or Ad5‐cTNT‐mir155‐scramble) was injected into the resection border zone of P1 mice after AR. At 7 days post‐resection (dpr), the resection border zone myocardium around the injection site was collected and used for Western blot, quantitative reverse transcription‐polymerase chain reaction (qRT‐PCR), and immunofluorescence staining analysis. Wheat germ agglutinin (WGA) staining was analyzed in the resection border zone myocardium around the injection site at 28 dpr. Using echocardiography assessed cardiac function was assessed at 1 and 28 dpr. Using Masson's trichrome staining, we measured the fibrotic area at 28 dpr.

After injection of AAV9‐cTNT‐HMGB2 or AAV9‐cTNT vector into the myocardium of P1 mice, the myocardium around the injection site was collected and analyzed by Western blot and qRT‐PCR analysis at P7, P14, P21, and P28. At P28, indexes of proliferation (pH3, Ki67, Aurora B, and EdU) were analyzed. The amount, size, nucleation, and ploidy of cardiomyocytes were detected by collecting the myocardial tissue around the injection site at P28. The histology of hearts and cardiac function were detected at P28.

The MI model was established in adult mice, and adeno‐associated virus (AAV9‐cTNT‐HMGB2 or AAV9‐cTNT vector) was injected into the infarct border zone. At 14 dpi, the infarct border zone was collected and used for Western blot, qRT‐PCR, and immunofluorescence staining analysis. Echocardiography was used to assess cardiac function at 1 and 28 dpi. WGA staining, the nucleation and ploidy of CMs, and the CM number were analyzed in the infarct border zone at 28 dpi. The fibrotic area was measured at 28 dpi by Masson's trichrome staining.

The MI model was induced in adult mice, followed by the injection of adeno‐associated virus (AAV9‐cTNT‐HIF‐1α or AAV9‐cTNT vector) into the infarct border zone. Indexes of proliferation (pH3, Ki67, and EdU) were detected at 14 dpi. The infarct border zone was collected for Western blot and immunofluorescence staining analysis at 14 dpi. Additionally, WGA staining was performed and analyzed at 28 dpi.

AAV9‐cTNT‐HMGB2 and Ad5‐cTNT‐mir30‐HIF‐1α (or AAV9‐cTNT‐mir155‐MTA2) were injected into the infarct border zone of adult mice right after MI. At 14 dpi, the infarct border zone myocardium around the injection site was collected and used for Western blot and immunofluorescence staining analysis. At 1 and 28 dpi, cardiac function was assessed. The fibrotic area was measured at 28 dpi by Masson's trichrome staining. The size of cardiomyocytes was detected in the infarct border zone myocardium around the injection site at 28 dpi.

### Construction of Recombinant Adenovirus 5 (Ad5), Adeno‐Associated Virus 9 (AAV9), and Plasmids

The adenovirus 5 (Ad5) carrying the HMGB2 coding sequence under the cTNT promoter (Ad5‐cTNT‐HMGB2) and adenovirus 5 (Ad5) carrying the HMGB2 shRNA for mouse HMGB2 under the cTNT promoter (Ad5‐cTNT‐mir155‐HMGB2) were constructed. They and their control vectors (Ad5‐cTNT vector, Ad5‐cTNT‐mir155‐scramble) were purchased from Genechem Company (Shanghai, China). The target sequence of shRNA for mouse HMGB2 was 5’‐ATAACGAGCTTTGTCGCTCTT‐3’.

The adenovirus 5 (Ad5) carrying the HIF‐1α shRNA for mouse HIF‐1α under the cTNT promoter (Ad5‐cTNT‐mir30‐HIF‐1α) was constructed. It and its control vectors (Ad5‐cTNT‐mir30‐scramble) were purchased from HANBIO Company (Shanghai, China). The target sequence of shRNA for mouse HIF‐1α was 5’‐GCTGACCAGTTACGATTGT‐3’.

The adenovirus 5 (Ad5) carrying the MTA2 shRNA for mouse MTA2 under the cTNT promoter (Ad5‐cTNT‐mir155‐MTA2) was constructed. It and its control vectors (Ad5‐cTNT‐mir155‐scramble) were purchased from Genechem Company (Shanghai, China). The target sequence of shRNA for mouse MTA2 was 5’‐CACACTCAAGAGGTCATTTAT‐3’.

The adeno‐associated virus 9 (AAV9) carrying the HMGB2 coding sequence under the cTNT promoter (AAV9‐cTNT‐HMGB2) and adeno‐associated virus 9 (AAV9) carrying the HIF‐1α coding sequence under the cTNT promoter (AAV9‐cTNT‐HIF‐1α) were constructed. They and their control vectors (AAV9‐cTNT vector) were purchased from Genechem Company (Shanghai, China).

The adeno‐associated virus 9 (AAV9) carrying the MTA2 shRNA for mouse MTA2 under the cTNT promoter (AAV9‐cTNT‐mir155‐MTA2) and control vector (AAV9‐cTNT‐mir155‐scramble) were purchased from Genechem Company (Shanghai, China). The target sequence of shRNA for mouse MTA2 was 5’‐CACACTCAAGAGGTCATTTAT‐3’.

Viral vectors (Ad5‐cTNT‐mir155‐HMGB2, Ad5‐cTNT‐HMGB2, Ad5‐cTNT‐mir155‐MTA2) and control viruses were diluted with phosphate‐buffered saline (PBS) and injected into the resection border zone using a 36G needle in P1 mice after AR. Each mouse received 1.5 × 10^7^ PFU of each type of adenovirus, administered in three equal injection sites around the resection border zone.

Viral vectors (AAV9‐cTNT‐HMGB2, AAV9‐cTNT‐HIF‐1α, Ad5‐cTNT‐mir30‐HIF‐1α, AAV9‐cTNT‐mir155‐MTA2) and control viruses were diluted with PBS and injected into the infarct border zone using a 36G needle in P56 mice after MI. Each mouse received 5 × 10^8^ PFU of each type of adeno‐associated virus, administered in three equal injection sites. Each mouse received 2.5 × 10^8^ PFU of each type of adenovirus, administered in three equal injection sites.

Plasmids carrying Flag‐HMGB2‐full‐length (210 amino acids), Flag‐HMGB2∆165 (165 amino acids), Flag‐HMGB2∆79 (79 amino acids), and HA‐MTA2‐full‐length were purchased from Genechem Company (Shanghai, China).

### Cell Isolation, Culture, and Intervention

Neonatal mouse cardiomyocytes (NMCMs) were isolated and extracted from 1‐day‐old C57BL/6J mice using methods previously reported.^[^ ^40^ ^]^ Briefly, after euthanizing the mice, ventricular myocardium was collected and washed in pre‐cooled phosphate‐buffered saline (PBS) to remove excess red blood cells. The ventricular myocardium was digested multiple times with a solution containing trypsin and collagenase type II for 6 min until the tissue achieved a single‐cell state. After incubation with Dulbecco's Modified Eagle Medium (DMEM) containing 10% fetal bovine serum (FBS) for 45 min, fibroblasts were separated by differential attachment, and the suspension was further centrifuged (3000 rpm, 30 min) to separate non‐CMs with Percoll solution. The NMCMs in the middle layer were collected and cultured for 48 h in DMEM with 10% horse serum (HS) and 5% FBS in a 37 °C incubator with 5% CO_2_, while the non‐CMs were cultured in DMEM with 10% FBS. The isolated cardiomyocytes were used across all relevant experimental analyses in this study, including Western blot, quantitative real‐time PCR, immunofluorescence staining, and other assays.

For exploring optimal MOI, NMCMs were infected with adenoviruses carrying eGFP‐tagged HMGB2 using multiplicities of infection (MOI) 10, 50, 100, and 200, respectively, for 24 h. To intervene in the expression of HMGB2 or MTA2 in NMCMs, corresponding adenoviruses (Ad5‐cTNT‐mir155‐HMGB2, Ad5‐cTNT‐HMGB2, Ad5‐cTNT‐mir155‐scramble, or Ad5‐cTNT vector) or plasmids (HA‐MTA2 or HA‐CON) were transfected into NMCMs. Similarly, Ad5‐cTNT‐mir155‐HMGB2, Ad5‐cTNT‐HMGB2, and the corresponding control vectors were transfected into non‐CMs to observe their effects on HMGB2 expression in non‐CMs.

As previously reported, juvenile C57BL/6J mice were used to isolate and extract cardiomyocytes.^[^ ^60^ ^]^ These mice included: a) P1+AAV9‐cTNT‐HMGB2 at 28 days, and b) P1+AAV9‐cTNT vector at 28 days.

As previously described, the Langendorff method was used to isolate and extract adult mouse cardiomyocytes (AMCMs) from 56‐day‐old C57BL/6J mice.^[^ ^61^ ^]^ The extracted AMCMs were cultured in M199 medium supplemented with bovine serum albumin (BSA), 2,3‐butanedione monoxime (2,3‐BDM), and penicillin‐streptomycin, and maintained in a 37 °C incubator with 5% CO_2._

The HEK293T cell line was obtained from Haixing Bioscience (Suzhou, China). The species of the cell line was verified through cytochrome B PCR and subsequent Sanger sequencing. Before conducting the experiment, the absence of Mycoplasma contamination was confirmed. The HEK293T cells were cultured in DMEM containing 10% FBS and 1% penicillin/streptomycin. Plasmids of HMGB2 and MTA2 were transfected following with lipofectamine 3000 reagent (Thermo Fisher, USA) according to the manufacturer's instructions.

### CM Number Quantification

NMCMs and juvenile cardiomyocytes were isolated from C57BL/6J mice as described above. For each hole of the 24‐well plate, eight to ten fields of view were randomly selected. And the number of CMs was counted using a microscope (Zeiss, Oberkochen, Germany). Counting the number of CMs per mm^2^ to calculate the CMs density. Cardiomyocyte counts were performed three times for each sample, with data collected from six hearts per group. To ensure accurate counting, only cells with clear vision were counted.

### Determining the Total Number of Nucleation and Ploidy of CMs

Cardiomyocytes were isolated from mice, and the isolated cardiomyocytes were fixed with 4% PFA at room temperature for 1 h. Hoechst 33342 (Thermo Fisher, H3570) and cardiac troponin T (cTNT) were utilized to label the CMs. The cell particles were thoroughly re‐suspended in 1mL of PBS, and 10 µL of the cell suspension was plated onto a slide. The amount of CMs was determined as the average of three independent counts per heart, conducted using a microscope (Zeiss, Oberkochen, Germany). To assess nucleation, each sample included approximately500 CMs, with six separate samples analyzed in each group. As previously described,^[^ ^50^, ^60^ ^]^ the Hoechst 33342 intensity of non‐CM nuclei within the same field was used to define the 2N ploidy of nuclei. The percentages of 2N, 4N, and >4N ploidy were calculated by normalizing the Hoechst 33342 intensity of CM nuclei to that of non‐CM nuclei. The following classifications were applied: 2N CM nuclei were defined as having a normalized integral pixel density between 0.5 and 1.5, 4N CM nuclei exhibited pixel density between 1.5 and 2.5, while nuclei with pixel density greater than 2.5 were classified as 4N+. Images of Hoechst 33342 signals were processed using Image J software, and the combined pixel density of individual nuclei in both CMs and non‐CMs was measured manually.

### Immunofluorescence Staining

Mouse hearts were collected at different time points, washed with PBS, fixed in 4% PFA for 24 h, and subsequently stored in 70% ethanol. Paraffin‐embedded myocardial sections (4 µm) underwent deparaffinization and acid antigen retrieval, followed by blocking and permeabilization using a solution containing 5% BSA and 0.1% Triton X‐100 for 2 h. The sections were then incubated with primary antibodies overnight at 4 °C. The following day, excess primary antibodies were removed by washing with phosphate‐buffered saline with Tween‐20 (PBST) for 30 min. Subsequently, the sections were incubated with secondary antibodies at room temperature for 2 h. Sections were washed three times in PBST, stained with Hoechst 33342 (Thermo Fisher, H3570) for 15 min to label nuclei. After an additional 30 min wash with PBST to eliminate unbound antibodies, images were observed and captured using a confocal microscope (Zeiss, Oberkochen, Germany).

In vitro, cardiomyocytes were fixed with 4% PFA for 15 min, followed by blocking and permeabilization using a solution containing 5% BSA and 0.1% Triton X‐100. The cardiomyocytes were then incubated with primary antibodies overnight at 4 °C. The following day, excess primary antibodies were washed off with PBST for 30 min. Subsequently, the sections were incubated with secondary antibodies at room temperature for 2 h. Sections were washed three times in PBST, stained with Hoechst 33342 (Thermo Fisher, H3570) for 15 min to label nuclei. After an additional 30 min wash with PBST to eliminate any unbound antibodies, images were captured using a confocal microscope (Zeiss, Oberkochen, Germany). The antibodies used for immunofluorescence staining in this study are detailed in Tables S2 and S3 (Supporting Information).

### EdU (5‐Ethynyl‐2′‐Deoxyuridine) Incorporation Assay

For in vitro cardiomyocyte staining, NMCMs were treated with 20 mm EdU for 2 h before fixation and subsequent staining. For in vivo myocardial tissue staining, EdU dye (5 mg/g body weight) was injected intraperitoneally for three days prior to myocardium collection from mice.

EdU staining was subsequently performed using a kit (Invitrogen, C10637) according to the manufacturer's instructions, while concurrently conducting immunofluorescence staining for additional components. Images were observed and captured using a confocal microscope (Zeiss, Oberkochen, Germany).

### Terminal Deoxynucleotidyl Transferase‐Mediated dUTP Nick End Labeling (Tunel) Assay

Cell apoptosis was assessed using the Tunel BrightRed Apoptosis Detection Kit (YSFluor 640) (40308ES60, Yeasen) according to the manufacturer's instructions. Briefly, cultured cells were fixed in 4% paraformaldehyde for 15 min, followed by permeabilization with 0.3% Triton X‐100 for 15 min at room temperature. The samples were then incubated with the Tunel labeling reaction solution for 60 min at 37 °C. Images were observed and captured using a confocal microscope (Zeiss, Oberkochen, Germany).

### Wheat Germ Agglutinin Staining and CM Size Quantification

Mouse hearts were collected at different time points, washed with PBS, and fixed in 4% PFA for 24 h before being stored in 70% ethanol. Paraffin‐embedded myocardial sections (4 µm) were subjected to deparaffinization and antigen retrieval in an acidic solution, followed by blocking and permeabilization using a solution containing 5% BSA and 0.1% Triton X‐100 for 2 h. The sections were then incubated with WGA at a dilution of 1:200 (Thermo Fisher, w32466) for 2 h. Sections were washed three times in PBS, stained with Hoechst 33342 (Thermo Fisher, H3570) for 15 min to label nuclei. Images were observed and captured using a confocal microscope (Zeiss, Oberkochen, Germany).

To ensure the accuracy of the quantitative analysis of cardiomyocyte size, 10 random fields were selected from each myocardial section for observation, measuring only the cross‐sectional area of the cardiomyocytes. The cross‐sectional area was quantified using ImageJ software.

### Measurement of Extracellular Acidification Rate (ECAR)

The ECAR of NMCMs was measured using the Seahorse XF Glycolysis Stress Test Kit (103020‐100, Agilent) in accordance with the manufacturer's instructions on a Seahorse XF flux analyzer 96. Briefly, NMCMs were seeded at a density of 60 000 cells per well in a 96‐well plate and subjected to the relevant experimental treatments. Following this, 10 mm glucose, 1.5 µm oligomycin (Oligo), and 50 µm 2‐deoxy‐glucose (2‐DG) were added. The glycolysis or glycolysis capacity of NMCMs was calculated as the maximum ECAR before or after adding oligomycin minus the last ECAR measurement before glucose addition, respectively. Data analysis was conducted using the software provided by the manufacturer (Seahorse Wave).

### Quantification of Intracellular Lactate Levels

Cardiomyocytes were extracted for the detection of intracellular lactate levels. Briefly, cardiomyocytes were seeded at a density of 5 × 10^6^ cells per well in a 6‐well plate. Lactate levels were measured using the L‐lactate assay kit (Abcam, ab65331) according to the manufacturer's instructions, with absorbance measured at a wavelength of 450 nm.

### Flow Cytometry

Prior to the assay, NMCMs were washed with PBS and subsequently centrifuged at 1000 rpm for 10 min. Following vortexing to ensure uniform mixing of the cardiomyocytes, 5 mL of pre‐cooled 70–80% ethanol was gradually added to the cell suspension, which was then incubated at −20 °C for 2 h. After incubation, the cells were washed with PBS and Stain buffer, followed by centrifugation at 1500 rpm for 10 min. The cells were resuspended in 0.5 mL of propidium iodide (PI)/RNase Staining Buffer and incubated at room temperature for 15 min for PI/RNase staining. Subsequently, the stained cells were analyzed using flow cytometry, and data were acquired using the BD FACSCalibur with CellQuest Pro software (BD, USA).

### RNA‐Sequencing (RNA‐Seq) and Data Analysis

NMCMs transfected with Ad5‐cTNT‐HMGB2 or Ad5‐cTNT vector were utilized for RNA‐sequencing (n = 3 per group). Total RNA was extracted and evaluated for purity and concentration using a NanoDrop 2000 spectrophotometer. The VAHTS Universal V5 RNA‐sequencing Library Prep kit was employed to construct the transcriptome library. The library was sequenced on the Illumina NovaSeq 6000 platform to generate 150 bp reads. Subsequent processing and pairing of the raw reads were conducted using fastp and Hisat2 software. Differential gene expression analysis was performed using the edgeR package (version 4.2.1), with genes defined as differentially expressed if adjusted P value < 0.05 and fold change > 1.2 or < 1/1.2.

The RNA‐sequencing dataset examining the dynamics of mouse cardiac development was obtained from the Gene Expression Omnibus (GSE213233, https://www.ncbi.nlm.nih.gov/geo/query/acc.cgi?acc = GSE213233). When adjusted P value < 0.05 and |Log_2_FC| > 2.5, genes were assigned as differentially expressed between adult mouse hearts (P56) and neonatal mouse hearts (P1).

Subsequent analyses, including Gene Ontology (GO), Kyoto Encyclopedia of Genes and Genomes (KEGG), and Gene Set Enrichment Analysis (GSEA), were conducted using the “ClusterProfiler” package (version 4.12.2) in R (version 4.4.1).

### Single‐Cell RNA‐Sequencing Analysis

The published single‐nucleus RNA sequencing data of cardiomyocytes from neonatal mouse hearts following injury were obtained from the Gene Expression Omnibus (GSE130699, https://www.ncbi.nlm.nih.gov/geo/query/acc.cgi?acc = GSE130699). For the analysis of the single‐nucleus RNA‐sequencing data, the Seurat V4 package was utilized. Initially, the data were loaded into Seurat objects using the Read10X function. To identify cardiomyocytes, the specific marker gene MYH6 was employed. Following this, the subset of cardiomyocytes was extracted, and a dot plot was used to visualize the expression levels of HMGB2 under different conditions.

### Tandem Mass Tag Labeling‐Based Quantitative Proteomics Analysis

Primary cardiomyocytes were isolated from P1 mice and 8‐week‐old (P56) mice for immunoprecipitation followed by mass spectrometry proteomics analysis (n = 3 per group). Briefly, the trypsin‐digested peptides were desalted using HLB columns and subsequently lyophilized. The peptides were then dissolved in 0.2 M TEAB and labeled with the Tandem Mass Tag reagent kit. High‐pH reversed‐phase HPLC separation was performed on an XBridge BEH130 C18 column (300 µm×150 mm, 1.7 µm, Waters). The resulting peptide powder was resuspended in 0.1% (v/v) formic acid and analyzed sequentially using the EASY‐nLC 1200 ultrahigh‐performance liquid chromatography system in conjunction with the Thermo Scientific Orbitrap Fusion Lumos Tribrid mass spectrometer. Secondary mass spectrometry data analysis was conducted using Proteome Discoverer (v2.4), while mouse protein sequence searches were conducted with MaxQuant software (version 1.3.0.5) against the Universal Protein Resource database, applying false discovery rate cut‐offs of 0.01 for proteins, peptides, and sites. A quantitative protein was defined as one that has been identified at least twice in three replicates. When adjusted P value < 0.05 and |Log_2_FC| > 2.5, proteins were identified as differentially expressed between AMCMs and NMCMs groups.

*Protein Extraction, Western Blot Analysis, and Co‐immunoprecipitation (CO‐IP) Analysis, and Immunoprecipitation Coupled with Mass Spectrometry (IP/MS) Analysis*

Myocardial tissue or cardiomyocytes were lysed using RIPA lysis buffer containing 0.5% EDTA, 0.1% protease inhibitor, and 1% phosphatase inhibitor. The prepared protein samples were separated by sodium dodecyl sulfate‐polyacrylamide gel electrophoresis (SDS‐PAGE) gel and subsequently transferred to a polyvinylidene fluoride membrane (Millipore). The membrane was blocked with 5% non‐fat milk at room temperature for 2 h, followed by overnight incubation with the primary antibody at 4 °C. The following day, the membrane was washed with Tris‐buffered saline with Tween‐20 (TBST) for 30 min to eliminate any unbound primary antibody, after which it was incubated with the secondary antibody at room temperature for 2 h. After an additional two‐hour washing with TBST, exposure imaging analysis was conducted. Relevant antibody information could be found in Tables S2 and S3 (Supporting Information).

For the co‐immunoprecipitation (CO‐IP) experiments, cardiomyocytes or HEK293T cells were collected and lysed. The corresponding primary antibody was added to the lysate and incubated overnight on a rotating shaker at 4 °C. The following day, rProtein A/G Magnetic IP/CO‐IP Kit beads (BK0004‐02, ACE Biotechnology, China) were added to the protein‐antibody mixture and incubated for an additional 6 h to ensure complete binding. After incubation, the bead mixture was washed three times with lysis buffer. Subsequently, the proteins bound to the beads were eluted and precipitated. The precipitated protein complex was then used for Western blot analysis to detect potential protein interactions. Following the CO‐IP assay, ubiquitination and acetylation levels were detected by Western blot with associated antibodies. Relevant antibody information could be found in Tables S2 and S3 (Supporting Information).

To identify potential interacting proteins with HMGB2, immunoprecipitated protein samples of HMGB2 were isolated using SDS‐PAGE gel, followed by silver staining (P0017S, Beyotime, China). The stained gels were subsequently prepared for mass spectrometry analysis to facilitate polypeptide and protein identification (Wuhan Genecreate Biological Engineering Co., Ltd).

### Quantitative Reverse Transcription‐Polymerase Chain Reaction (qRT‐PCR) Assay

Total RNA was extracted from cells and cardiac tissue using TRIzol reagent (Thermo Fisher Scientific, Waltham, USA). Subsequently, RNA was reverse transcribed into complementary DNA (cDNA) utilizing the HiScript III RT SuperMix (R323‐01, Vazyme Biotech, Nanjing, China). Quantitative reverse transcription polymerase chain reaction (qRT‐PCR) was conducted employing the SYBR Green method (Q131‐02, Vazyme Biotech, Nanjing, China), with 18S ribosomal RNA serving as the control gene. The relevant primers utilized in this study are detailed in Table S4 (Supporting Information).

### Statistical Analysis

Statistical analyses were conducted using GraphPad Prism 8.0 software (San Diego, CA, USA). Data were presented as mean ± standard deviation (SD). The normality of the data distributions was assessed using the Shapiro‐Wilk test, and all datasets followed a normal distribution. To compare two groups, an unpaired two‐tailed *t*‐test was employed. For comparisons among multiple groups, one‐way ANOVA followed by Tukey's Multiple Comparison Test or two‐way ANOVA followed by Tukey's Multiple Comparison Test was employed. Statistical difference was considered significant when the results had with P value < 0.05.

## Conflict of Interest

The authors declare no conflict of interest.

## Author Contributions

L.‐H.Z. and L.‐F.G. contributed equally to this work. L.Z. conducted both in vitro and in vivo experiments, analyzed the data, and wrote the manuscript. L.G. carried out the in vitro experiments and performed data analysis. Y.C. and P.J. conducted certain in vivo experiments and participated in data analysis. T.Y. and Y.H. contributed to various in vitro experiments. Y.B. contributed to various in vivo experiments. X.Z. performed specific bioinformatics analyses. C.D. and S.W. contributed to various in vivo experiments. T.S., J.C., H.W., and Q.W. performed specific bioinformatics analyses. L.X. was responsible for supervision. Y.Z. contributed to the statistical analysis. Y.J. was responsible for supervision. L.W. (corresponding author) was responsible for the overall study design and supervision, as well as manuscript editing.

## Supporting information



Supporting Information

## Data Availability

The data that support the findings of this study are available from the corresponding author upon reasonable request.
